# Neurogenesis drives hippocampal formation-wide spatial transcription alterations in health and Alzheimer's disease

**DOI:** 10.3389/frdem.2025.1546433

**Published:** 2025-04-16

**Authors:** Zachery D. Morrissey, Pavan Kumar, Trongha X. Phan, Mark Maienschein-Cline, Alex Leow, Orly Lazarov

**Affiliations:** ^1^Graduate Program in Neuroscience, University of Illinois Chicago, Chicago, IL, United States; ^2^Department of Psychiatry, University of Illinois Chicago, Chicago, IL, United States; ^3^Department of Anatomy and Cell Biology, University of Illinois Chicago, Chicago, IL, United States; ^4^Research Informatics Core, University of Illinois Chicago, Chicago, IL, United States; ^5^Department of Biomedical Engineering, University of Illinois Chicago, Chicago, IL, United States

**Keywords:** Alzheimer's disease, neurogenesis, spatial transcriptomics, hippocampus, adult hippocampal neurogenesis

## Abstract

The mechanism by which neurogenesis regulates the profile of neurons and glia in the hippocampal formation is not known. Further, the effect of neurogenesis on neuronal vulnerability characterizing the entorhinal cortex in Alzheimer's disease (AD) is unknown. Here, we used *in situ* sequencing to investigate the spatial transcription profile of neurons and glia in the hippocampal circuitry in wild-type mice and in familial AD (FAD) mice expressing varying levels of neurogenesis. This approach revealed that in addition to the dentate gyrus, neurogenesis modulates the cellular profile in the entorhinal cortex and CA regions of the hippocampus. Notably, enhancing neurogenesis in FAD mice led to partial restoration of neuronal and cellular profile in these brain areas, resembling the profile of their wild-type counterparts. This approach provides a platform for the examination of the cellular dynamics in the hippocampal formation in health and in AD.

## 1 Introduction

Adult-born neurons (ABNs) are generated from neural stem cells that reside in the sub-granular zone (SGZ) of the dentate gyrus (DG) of the hippocampus. As they mature, ABNs incorporate into the granule cell layer of the DG. These neurons extend dendrites into the outer molecular layer of the DG and form synapses with neurons in layer II of the entorhinal cortex (ECx-II), while their axons form synapses with neurons in CA3 (Toda et al., 2019). ABNs play a role in hippocampal function, particularly learning and memory tasks, such as spatial navigation and recognition memory (Deng et al., 2009; Jessberger et al., 2009; Sahay et al., 2011; Van Praag et al., 2002). However, whether neurogenesis regulates the profile of neurons and glia in the hippocampal formation is not known.

Hippocampal neurogenesis is reduced in aging and deficient in Alzheimer's disease (AD) patients and mouse models (Boldrini et al., 2018; Demars et al., 2010, 2013; Lazarov et al., 2010; Moreno-Jiménez et al., 2019; Tobin et al., 2019). AD is characterized by progressive memory loss and cognitive deterioration due to dysfunction of vulnerable neurons (Lazarov and Hollands, 2016). Vulnerability in the human brain develops in ECx-II, followed by the CA1 of the hippocampus and, subsequently, other cortical areas (Braak and Braak, 1996). Synaptic loss is hypothesized to be an early manifestation of this vulnerability that precedes neuronal cell death. Thus, understanding the cross-talk between vulnerable neurons and their synaptic connections may reveal new information on the cause of synaptic loss. The effect of neurogenesis on the profile of neurons in the entorhinal cortex (EC) is of particular importance. Understanding the dynamics between levels of neurogenesis and the cellular profile of EC neurons may provide critical information about the role of new neurons in hippocampal function as well as in resilience or pathology. Previously, we have shown that augmenting neurogenesis in a mouse model of Alzheimer's disease rescues the neuronal memory ensemble in the DG and restores learning and memory, suggesting an effect on hippocampal function in FAD (Mishra et al., 2022). Using *in situ* sequencing spatial transcriptomics, combined with a viral engram reporter, we examined the differential gene expression of mature and immature neurons that participated in the engram. We found genes involved in FAD phenotype (*App, Apoe, Adam10*), neuronal regulation (*Bdnf, Mapk3*), calcium signaling (*Camk2a*), and neurogenesis (*Neurod1*), amongst others, were differentially expressed in wild-type and FAD mice with enhanced neurogenesis compared to FAD mice. Notably, in FAD mice with enhanced neurogenesis, the overall transcription profile of mature and immature neurons appeared more similar to wild-type mice compared to the FAD mice, suggesting that enhanced neurogenesis may, in part, restore the transcription profile of neurons similar to the wild-type state.

Here, we tested whether deficits in neurogenesis altered the transcription profile of mature neurons and glia in the hippocampal formation and whether augmenting neurogenesis in FAD rescues their profile. Previously, Roussarie et al. (2020) showed that the molecular identity of AD-vulnerable and -resistant neurons is largely conserved between mouse and human, providing evidence for the validity of mouse models in studying selective neuronal vulnerability. We used *in situ* sequencing (spatial transcriptomics) to investigate the transcription profile of neurons and glia in the hippocampal circuitry of a mouse model of AD with varying levels of neurogenesis. We used the *Nestin-CreER*^*T2*^*;Bax*^*fl/fl*^ (NB) mouse model system (Sahay et al., 2011) to inducibly increase the survivability of adult-born neurons upon injection of tamoxifen, effectively increasing neurogenesis. By crossing NB mice with the 5XFAD mouse model of familial Alzheimer's disease (Oakley et al., 2006) (*Nestin-CreER*^*T2*^*;Bax*^*fl/fl*^*;5XFAD*, abbreviated NBF), we could study the effects of healthy and FAD mice with and without enhanced neurogenesis. We observed that the cellular profile of the hippocampal formation of FAD mice was vastly different than wild-type mice, and that the profile of cells in an FAD mouse with elevated levels of neurogenesis (tamoxifen-treated NBF, abbreviated T–NBF) had a significant correlation with the NB profile. While the total number of neurons in the CA1, CA3, and EC was similar in NB and NBF mice, there were significant changes in the profile of both excitatory and inhibitory neurons, suggesting that the imbalance in hippocampal circuitry in AD stems, at least in part, from an altered neuronal profile rather than neuronal loss. Interestingly, the largest changes in differentially expressed genes (DEGs) were observed across cell types in the ECx. Together, the findings of this study provide a resource for the understanding of cellular dynamics in the hippocampal formation.

## 2 Results

### 2.1 Spatial transcriptome profile in the hippocampal formation

**Figure 1:**
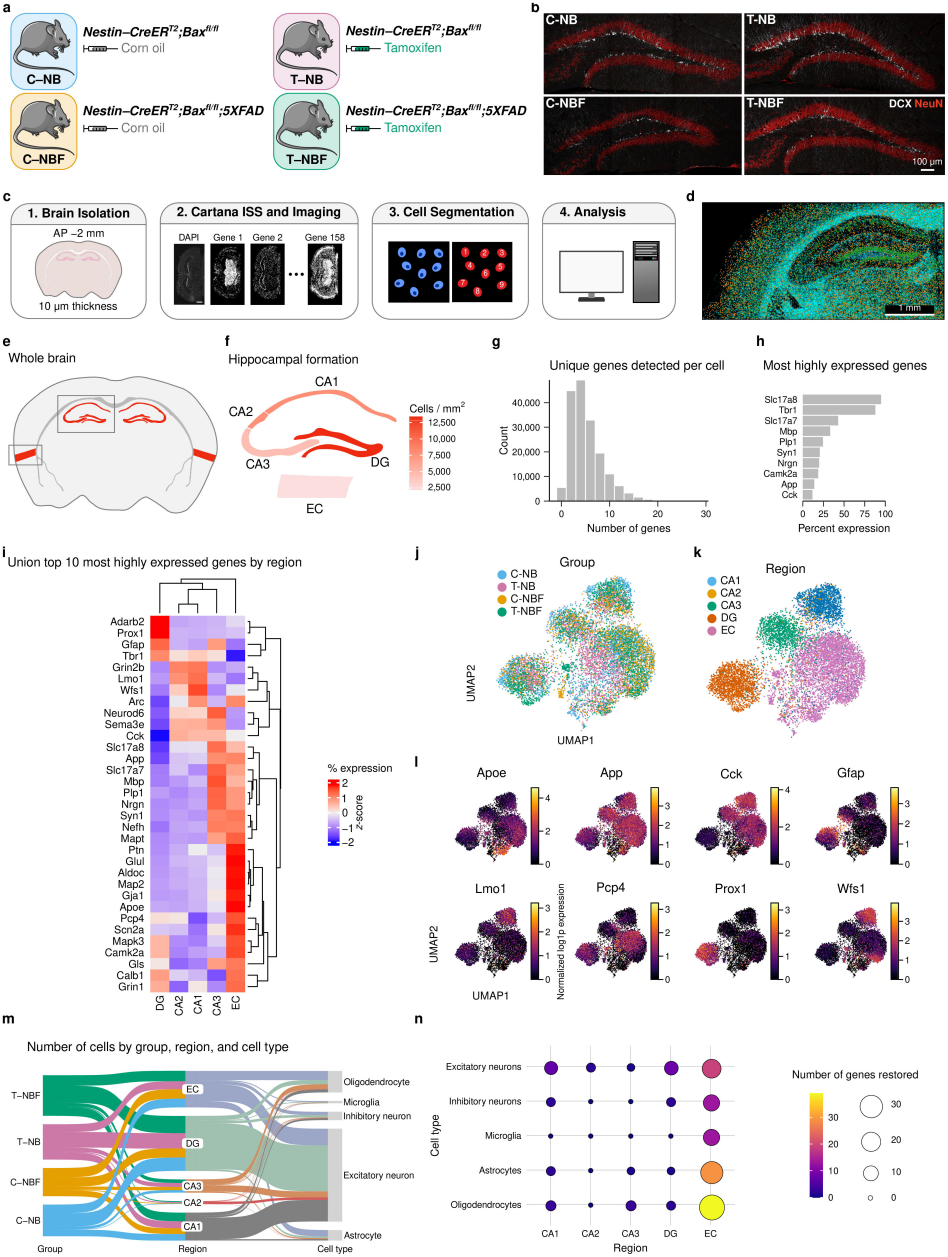
Transcription profile of the hippocampal formation. **(a)** Mouse genotypes. C–NB: *Nestin-CreER*^*T2*^*;Bax*^*fl/fl*^ injected with corn oil (vehicle). T–NB: *Nestin-CreER*^*T2*^*;Bax*^*fl/fl*^ injected with tamoxifen. C–NBF: *Nestin-CreER*^*T2*^*;Bax*^*fl/fl*^*;5XFAD* injected with corn oil. T–NBF: *Nestin-CreER*^*T2*^*;Bax*^*fl/fl*^*;5XFAD* injected with tamoxifen. **(b)** Immunofluorescence staining of coronal mouse dentate gyrus (DG) for each group. Red: NeuN; white: DCX. Scale bar indicates 100 μm. **(c)** Experiment design. 10 μm coronal sections were isolated from each animal and processed for Cartana *in situ* sequencing, cell segmentation, and analysis. **(d)** Representative spatial scatter plot of spots for a sample of genes including *Prox1, Neurod6, Rbfox3* (green, neuron-related), *Mbp, Plp1* (cyan, oligodendrocyte-related), *Acqp4, Aldoc* (orange, astrocyte-related), and *Laptm5, Itgam* (blue, microglia-related). Atlas schematic of approximate coronal slice location used for analysis. The hippocampal formation including entorhinal cortex (EC) is highlighted in red. **(f)** Cells per mm^2^ for each region of the hippocampal formation. The color indicates the average across all samples for each region. **(g)** Histogram of the number of unique genes expressed per cell. **(h)** Bar plot of the 10 most highly expressed genes across all cells. **(i)** Heatmap of the union top ten (32 unique) most highly expressed genes by region. Color indicates the percent expression (column) *z*-score for each gene in each region. **(j, k)** UMAP embedding of binned gene spot counts. Each point represents a 50 μm2 area of tissue. Color indicates either group **(j)** or region **(k)**. **(l)** UMAP representation of a selection of genes shown in **(i)**. Color indicates normalized ln(1+*x*) (i.e., log1p) gene expression. **(m)** Sankey diagram depicting the quantity of cell types in each region for each group. **(n)** Dot plot of the number of genes restored in the different cell types in each region of the hippocampal formation, in T–NBF, resembling their expression in C–NB, rather than C–NBF. The size and color of each dot indicates the number of restored genes in T–NBF for each cell type and region.

We sought to test the hypothesis that the level of hippocampal neurogenesis affects the profile of the cellular environment in the hippocampal formation in health and AD. We used 4.5-month-old female *Nestin-CreER*^*T2*^*;Bax*^*fl/fl*^ (NB) or *Nestin-CreER*^*T2*^*;Bax*^*fl/fl*^*;5XFAD* (NBF) mice that were treated with either corn oil (C–NB or C–NBF) or tamoxifen (T–NB or T–NBF) at 1 month of age (Figure 1a). After injection of tamoxifen, Cre-LoxP-mediated deletion of the apoptotic gene *Bax* in nestin-positive neural stem cells effectively increased the survival of immature neurons (DCX^+^ NeuN^+^ cells) in the DG (*n* = 2 biological replicates, Figure 1b). We have previously quantified DCX^+^ cells after *Bax* ablation at this timepoint provided in Figure 1, Supplementary Figures S1, S2 of Mishra et al. (2022). Briefly, the C–NBF mice have approximately half the number of DCX^+^ cells compared to the C–NB group. Injection of tamoxifen results in nearly double the number of DCX^+^ cells for both the T–NB and T–NBF groups. Coronal sections from these mice containing the dorsal hippocampus were isolated for spatial transcriptomics processing (Figures 1c, d, see Methods for details).

The gene expression profile of cells in the principal layers of the CA1–3, DG, and entorhinal cortex (EC) of these mice were analyzed by *in situ* sequencing using a panel of 158 genes (Figure 1e, Supplementary Figure S1). Of these regions, the cell density was the highest in the DG (Figure 1f). A group comparison revealed that the number of cells per mm^2^ within each region and the area of the DG across groups was comparable (Kruskal-Wallis test, χ^2^(3) = 4.419, *p* = 0.22, Supplementary Figures S1a–c).

Examination of gene expression in the hippocampus and entorhinal cortex across all cells revealed that the median number of unique genes detected was five (Figure 1g). The top ten most highly expressed detected genes included *Slc17a8, Tbr1, Slc17a7, Mbp, Plp1, Syn1, Nrgn, Camk2a, App*, and *Cck* (Figures 1h, i). (Detailed counts and relative expression of each of the 158 genes is shown in Supplementary Figure S1). Next, we performed UMAP on binned spot counts of the hippocampal formation to create a low-dimensional embedding of the data and observed clustering of gene expression by group (Figure 1j) and especially by region (Figure 1k, Supplementary Figure S2). Within each region, *Slc17a8, Tbr1, Slc17a7*, and *Mbp* were consistently the most highly expressed (Supplementary Figures S2d–h). Comparing across the regions, *Adarb2, Prox1, Gfap*, and *Tbr1* were most relatively highly expressed in the DG, whereas *Grin2b, Lmo11*, and *Wfs1* were most relatively highly expressed in the CA1 and CA2, *Neurod6, Sema3e*, and *Cck*, were more relatively highly expressed in the CA3, *Slc17a8, App, Slc17a7, Mbp, Plp1, Nrgn, Syn1, Nefh*, and *Mapt* were more relatively highly expressed in the CA3 and EC, and *Ptn, Glul, Aldoc, Map2, Gja1, Apoe, Pcp4, Scn2a, Mapk3, Camk2a, Gls, Calb1*, and *Grin1* were uniquely higher in the EC. A selection of these genes is shown in the UMAP embedding space in Figure 1l showing their region localization. Next, we annotated cells based on known marker gene expression for excitatory and inhibitory neurons, oligodendrocytes, astrocytes, and microglia (Figure 1m). The majority of cells across all regions were excitatory neurons, followed by oligodendrocytes, astrocytes, inhibitory neurons, immature neurons, and microglia (Figure 1m, Supplementary Table S1). Full details of relative gene expression by region and cell type are shown in Supplementary Figure S3. Finally, our previous study suggested that augmentation of neurogenesis in FAD mice restored the profile of DG granule neurons recruited into the memory circuit following learning (Mishra et al., 2022). Thus, we asked whether augmentation of neurogenesis restores, at least in part, the transcription profile of neurons and glia in other regions of the hippocampal formation. This analysis revealed that enhancing neurogenesis restored genes in neurons, glia, and microglia, across all regions of the hippocampal formation (Figure 1n).

### 2.2 Restored genes in neuron profile in T–NBF resembling C–NB

**Figure 2:**
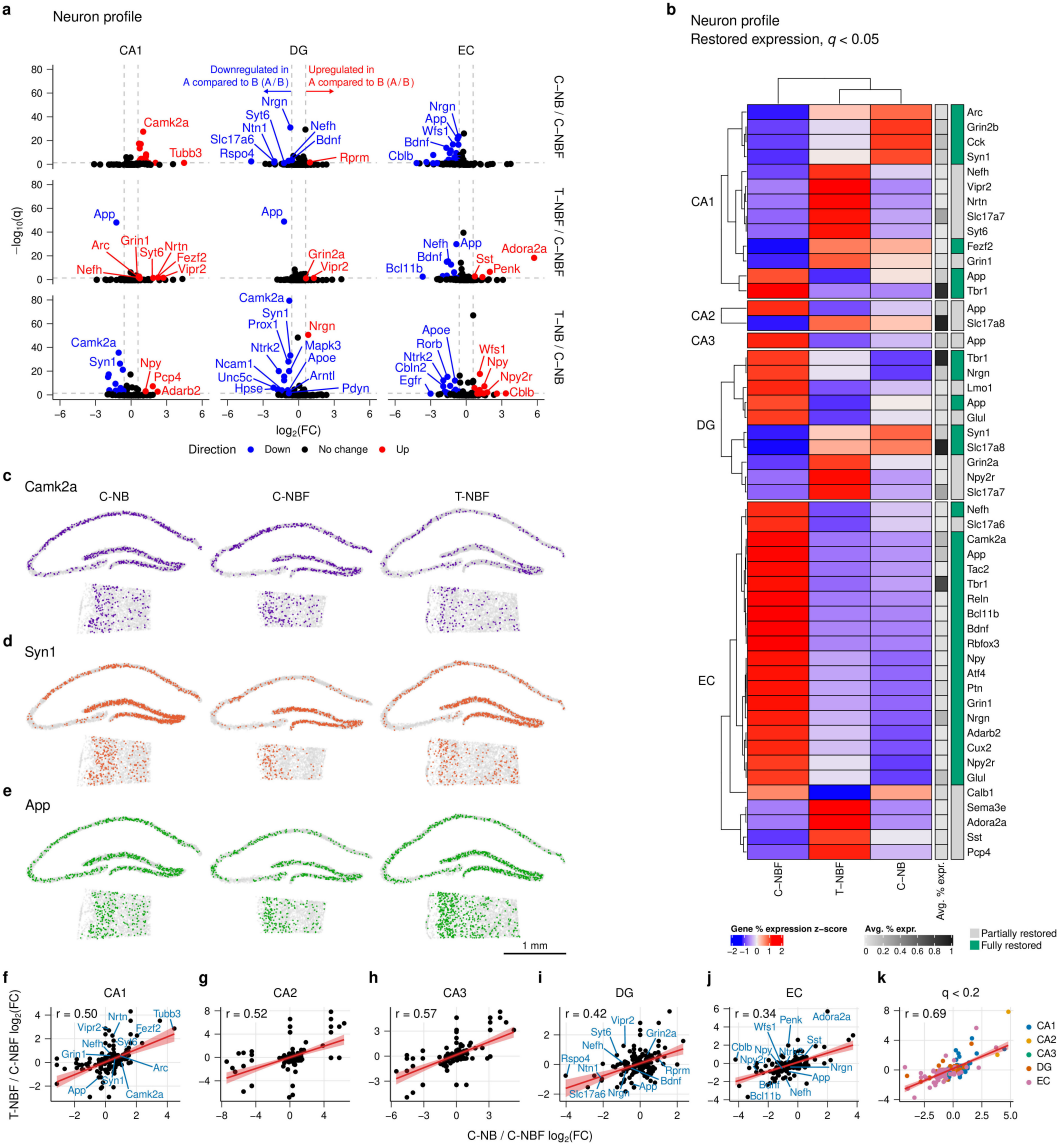
Neuron profile differential gene expression analysis. **(a)** Volcano plot of differentially expressed genes in neurons in each region for C–NB/C–NBF, T–NBF/C–NBF, and T–NB/C–NB. Differential gene expression was performed using FET and Benjamini-Hochberg's FDR was applied to correct for multiple comparisons. Red points indicate upregulated genes and blue points indicate downregulated genes. Vertical reference lines indicate log_2_(1.5) and log_2_(1/1.5) for up- and downregulation cutoffs, respectively. Horizontal reference lines indicate −log(0.05). **(b)** Heatmap of restored expression profile in each region for neuron (*q* < 0.05). Color indicates (row) *z*-scored percent expression of mature neurons that were positive for that gene. Grayscale bar indicates the average percent expression of each gene across all neurons in each region. Green indicates genes that were fully restored. **(c–e)** Representative scatter plot of restored genes *Camk2a* **(c)**, *Syn1* **(d)**, and *App* **(e)**. **(f–j)** Linear regression of log_2_(*FC*) consistency between C–NB and T–NBF relative to C–NBF for each region across all genes. Each point represents a gene. Line represents linear model fit. **(k)** Linear regression of most significant genes. Each point represents a gene with *q* < 0.2 for at least one comparison. Color indicates region. Line represents model fit.

To examine the neurogenesis-dependent cell profile in each cell type and region, we first examined the profile of neurons in each region and group. To compare the neuronal profile in regions of the hippocampal formation, we used Fisher's exact test (FET) to perform pairwise differential gene expression for each gene in mature neurons in the CA1–3, DG, and EC in C–NB relative to C–NBF (C–NB/C–NBF), and T–NBF relative to C–NBF (T–NBF/C–NBF). Interestingly, comparing differentially expressed genes (DEGs) in the C–NB/C–NBF and T–NBF/C–NBF comparisons revealed similar trends. Specifically, among the 158 genes across all five regions, we observed 20 genes upregulated and 47 genes downregulated in C–NB/C–NBF, 13 genes upregulated and 16 genes downregulated in T–NBF/C–NBF, and 21 genes upregulated and 56 genes downregulated in T–NB/C–NB (Figure 2a, Supplementary Figure S4). Of the five regions, the EC and CA1 had the largest number of differentially expressed genes. In the EC, nearly all the statistically significant genes were upregulated in C–NBF compared to C–NB and T–NBF, with the lowest *q* values and highest log_2_(fold change) (FC) including *Nrgn, App, Wfs1, Glul, Bdnf, Rbfox3, Gls, Atf4, Npy2r*, and *Camk4*. In T–NBF/C–NBF, the most significant genes that were downregulated included *App, Nefh, Bdnf, Rbfox3, Atf4, Reln, Tac2*, and *Bcl11b*, and those that were upregulated included *Adora2a, Penk, Sema3e*, and *Sst*. In the CA1, there was a similar trend in the fold change direction between C–NB/C–NBF and T–NBF/CNBF. Most of the significant genes with large fold changes were downregulated in C–NBF, including *Camk2a, Cck, Grin2b, Syn1, Scn2a, Ptn, Arc, Per1, Gabra1*, and *Hdac2*. In the T–NBF/C–NBF comparison, *Grin1, Arc, Fezf2, Nrtn, Vipr2, Syt6*, and *Nefh* were downregulated in C–NBF. Notably, *App* was downregulated in T–NBF in the CA1, DG, and EC. For T–NB/C–NB, the most significant upregulated genes included *Pcp4* (CA1), *Npy* (EC), *Wfs1* (EC), *Nrgn* (DG), and *Gls* (EC), and the most significant downregulated genes inlcuded *Syn1* (CA3), *Camk2a* (CA1), *Prox1* (DG), *Camk2a* (DG), and *Syn1* (DG).

These results support the notion that augmentation of neurogenesis restored the transcription profile of both immature and mature neurons in FAD mice. Coupled with the observation that augmentation of neurogenesis restores, at least in part, the transcription profile of neurons in other regions of the hippocampal formation (Figure 1n), we examined the genes that were restored in neurons. To do so, we identified which genes were statistically significantly altered (*q* < 0.05) in T–NBF/C–NBF that also showed the same fold change (FC) direction in C–NB relative to C–NBF, i.e., genes that were “restored” in T–NBF similar to C–NB (Figure 2b); among those genes, we further defined genes as “fully restored” if the fold change was statistically significant for both comparisons. We found that 50 of the 158 genes met this criterion across the five regions (EC: 24; CA1: 13; DG: 10; CA2: 2; CA3: 1; Figures 2b–e). Restored genes with the highest percent expression included *Slc17a8* (DG, CA2), *Tbr1* (CA1, DG, EC), *Slc17a8* (CA1, DG), *Nrgn* (EC), *Camk2a* (EC), and *Cck* (CA1). Notably, *App* was “restored” in all five regions in the T–NBF (Figures 2b, e). The profile of neurons in the DG of C–NB and C–NBF revealed ten genes that were restored: *Tbr1, Nrgn, Lmo1, App* and *Glul* were significantly higher in the C–NBF mice, while *Syn1* and *Slc17a7* were increased compared to C–NBF. *Grin2a, Npy2r*, and *Slc17a7* were increased in the T–NBF group.

A similar trend was observed in neurons in the EC. Many genes were altered in C–NBF compared to C–NB, while their expression was comparable in C–NB and T–NBF. *App, Nrgn, Rbfox3*, and *Bdnf* were altered in C–NBF, while comparable expression was observed in C–NB and T–NBF. Yet, several genes were differentially expressed in T–NBF, specifically, *Calb1, Sema3e, Adora2a, Sst*, and *Pcp4*. A few genes were restored in the CA2 and CA3 regions. In both regions, *App* was upregulated in neurons in the C–NBF compared to C–NB and T–NBF. *Slc17a8* was upregulated in neurons in the CA2 region in both C–NB and T–NBF compared to C–NBF.

Taking these results into consideration, we hypothesized that enhanced neurogenesis in FAD rescued the gene expression profile comparable to wild-type. To answer this, we quantified how consistent the T–NBF profile was to the C–NB profile. Within each region, we performed linear regression of the log_2_(FC) for each gene between the C–NB/C–NBF and T–NBF/C–NBF comparisons (Figures 2f–j). We observed a statistically significant fit of the model within each region (Supplementary Table S3). To better account for the statistical significance of each gene, we also filtered the gene list to only include genes with Benjamini-Hochberg FDR *q* < 0.2. We observed a statistically significant fit (adjusted R^2^ = 0.48, *r* = 0.69, *p* < 0.001; Figure 2k, Supplementary Table S3). Taken together, these results suggest that there was a consistent change in the gene expression profile between C–NB and T–NBF relative to C–NBF, suggesting that augmentation of neurogenesis in 5XFAD rescues the transcription profile of neurons in regions of the hippocampal formation beyond the DG.

### 2.3 Profile of excitatory and inhibitory neurons in the hippocampal formation in FAD

**Figure 3:**
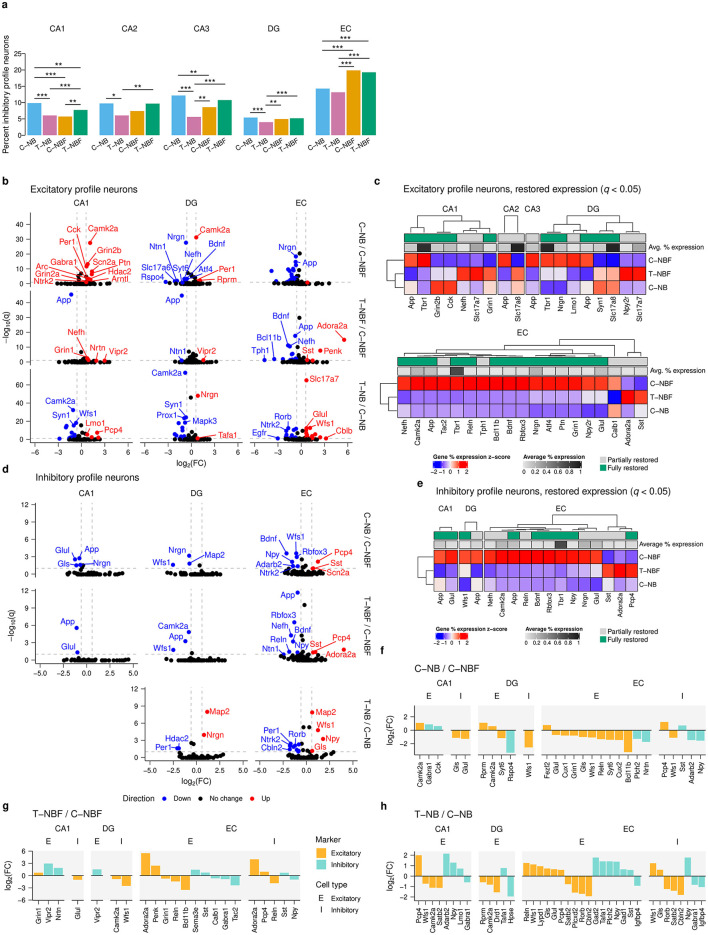
Excitatory and inhibitory neuron transcription profile analysis. **(a)** Bar plot of percent inhibitory neurons of total neurons in each region for each group. Each bar represents the percentage of inhibitory neurons relative to the total number of mature neurons, pooled within group. Statistics were performed using Fisher's exact test (FET) to compare the abundance of cells between each pair of groups. Significance code: ^***^p < 0.001; ^**^p < 0.01; ^*^p < 0.05. **(b)** Volcano plot of excitatory profile neurons in the CA1, DG, and EC for each comparison. Differential gene expression was performed using FET and Benjamini-Hochberg's FDR was applied to correct for multiple comparisons. Red points indicate upregulated genes and blue points indicate downregulated genes. Vertical reference lines indicate *log*_2_(1.5) and *log*_2_(1/1.5) for up- and downregulation cutoffs, respectively. Horizontal reference lines indicate −*log*(0.1). **(c)** Heatmap of restored expression profile in each region for excitatory profile neurons (*q* < 0.05). Color indicates (column) *z*-scored percent expression of mature neurons that were positive for that gene. Grayscale average percent expression bar indicates the average percent expression of that gene across all mature neurons in each region. Green indicates genes that were fully restored. **(d)** Volcano plot for inhibitory profile neurons in the CA1, DG, and EC. **(e)** Heatmap of restored expression profile in each region for inhibitory profile neurons (*q* < 0.05). **(f–h)** Comparison of excitatory and inhibitory marker expression in excitatory and inhibitory profile neurons for T–NB/C–NB **(f)**, and T–NBF/C–NBF **(g)**, and C–NB/C–NBF **(h)** comparisons. Color indicates excitatory/inhibitory marker. Orange: excitatory; teal: inhibitory. Bars represent log_2_(FC). E: excitatory profile neuron; I: inhibitory profile neuron.

Increasing evidence suggests that there is excitation-inhibition (E/I) imbalance in AD (Targa Dias Anastacio et al., 2022; Ghatak et al., 2019). However, the mechanism that causes an imbalance is not fully understood. To gain an insight into this issue, we asked whether there were alterations in the gene profile of excitatory and inhibitory neurons in the hippocampal formation of these mice. Excitatory and inhibitory profiles were determined based on the presence of known marker gene expression. There was a reduced proportion of inhibitory profile neurons in the CA1 and CA3 of the C–NBF group compared to the C–NB and T–NBF groups (Figure 3a, Supplementary Table S4). In contrast, the proportion of inhibitory profile neurons in the EC was increased in both the C–NBF and T–NBF groups compared to the C–NB (Figure 3a). Interestingly, the percentage of neurons with inhibitory profile in the T–NB group was significantly lower in the CA1, CA2, CA3, and DG relative to C–NB, with the largest effect size in the CA3 (C–NB/T–NB log_2_(odds ratio) = 1.22), suggesting that increased neurogenesis modulates the excitatory-inhibitory ratio even in the healthy condition, albeit in a distinct manner compared to the alterations in the diseased brain.

To investigate which genes underlie changes in neuronal profile, we analyzed the log_2_(FC) of genes detected in excitatory and inhibitory neurons between the C–NB/C–NBF and T–NBF/C–NBF (Figures 3b–e). In excitatory neurons, 21 genes were upregulated and 47 genes were downregulated. In the CA1, for C–NB/C–NBF, most of the significantly differentially expressed genes were downregulated in C–NBF, the most significant of which included *Camk2a, Grin2b, Cck, Scn2a*, and *Ptn* (Figure 3b). In T–NBF/C–NBF, *App* was downregulated in T–NBF while *Grin1* and *Nefh* were upregulated (Figure 3b). Similar to the mature neurons, the EC showed the largest number of differentially expressed genes for both excitatory (Figure 3b) and inhibitory neurons (Figure 3d), with C–NB/C–NBF showing mostly upregulated genes in C–NBF, and T–NBF/C–NBF showing mostly downregulated genes. In excitatory neurons, *Nrgn, App, Glul, Wfs1*, and *Atf* were upregulated in C–NBF relative to C–NB, and *App, Bdnf* , and *Nefh* were downregulated, and *Adora2a* and *Penk* were upregulated in T–NBF relative to C–NBF (Figure 3b). Similarly, in T–NB/C–NB, most genes were differentially expressed in the EC. There were 17 genes upregulated and 15 genes downregulated, with the most signficant of which included *Slc17a7, Wfs1, Glul, App*, and *Map2* that were upregulated and *Rorb, Syn1, Apoe, Ntrk2*, and *Cbln2* that were downregulated. Examination of genes that were restored in 5XFAD mice following enhanced neurogenesis in T–NBF mice revealed an excitatory gene expression profile in the CA2, CA3, DG, and EC that was markedly different in the C–NBF group compared to the C–NB counterpart, while the profile of the T–NBF group seemed comparable to the C–NB one. The largest number of restored genes was observed in the EC. *Tbr1, Slc17a8, Camk2a*, and *Slc17a7* were among the highest expressing genes restored in excitatory neurons (Figure 3c).

In inhibitory neurons, the CA1, DG, and EC showed the most differentially expressed genes (Figure 3d). In the CA1, *App, Glul, Nrgn*, and *Gls* were downregulated in C–NB/C–NBF, while *App* and *Glul* were downregulated in T–NBF/C–NBF. In the DG, *Nrgn, Map2*, and *Wfs1* were downregulated in C–NB/C–NBF, and *Camk2a, App*, and *Wfs1* were downregulated in T–NBF/C–NBF. Finally, in the EC, the top differentially expressed genes downregulated in C–NB/C–NBF included *Bdnf, Wfs1, Rbfox3, Pcp4*, and *Npy*. In T–NBF/C–NBF, among the top differentially expressed genes *App, Rbfox3, Nefh, Bdnf, Reln*, and *Npy* were downregulated, while *Adora2a, Pcp4*, and *Sst* were upregulated. In T–NB/C–NB, most of the differentially expressed genes were also in the EC. *Map2, Wfs1, Npy*, and *Gls* were upregulated and *Per1, Gabra1, Rorb, Ntrk2, Satb2, Cbln2, Igfbp4, Apoe*, and *Arc*.

Similar to the excitatory neuron transcription profile, examination of the genes that were rescued following augmentation of neurogenesis in inhibitory neurons in the T–NBF mice revealed a group of genes that were markedly different in the C–NBF group compared to the C–NB counterpart, and were partially or fully rescued in the T–NBF mice. *Tbr1, Camk2a, Glul*, and *Nrgn* were among the highest expressing restored genes in inhibitory neurons (Figure 3e).

Next, we examined alterations in excitatory and inhibitory markers exclusively (Figures 3f–h). In C–NB/C–NBF (Figure 3f), most excitatory markers in the EC were downregulated. In both C–NB and T–NBF relative to C–NBF, the excitatory markers *Bcl11b, Reln*, and *Grin* were downregulated in excitatory neurons (Figures 3f, g). In inhibitory neurons, *Pcp4, Npy*, and *Sst* showed similar trends in C–NB and T–NBF compared to C–NBF. *Adora2a* was upregulated in both excitatory and inhibitory neurons in the EC of T–NBF compared to C–NBF (Figure 3g). In excitatory neurons in the CA1, excitatory markers, such as *Camk2a* in C–NB and *Grin1* in T–NBF, were higher compared to C–NBF (Figures 3f, g). Likewise, inhibitory markers, such as *Cck* and *Gabra1* in C–NB and *Nrtn* and *Vipr2* in T–NBF, were increased compared to C–NBF. The excitatory marker *Glul* was downregulated in inhibitory neurons in both C–NB and T–NBF compared to C–NBF. In the DG, *Camk2a* was upregulated in both excitatory neurons in C–NB and inhibitory neurons in T–NBF compared to C–NBF. Likewise, the excitatory marker *Wfs1* was downregulated in C–NB and T–NBF compared to C–NBF. In the T–NB/C–NB comparison (Figure 3h), there were more changes in excitatory neurons compared to inhibitory neurons, with most changes taking place in the CA1 and EC. *Npy* was consistently increased in both the CA1 and EC, *Satb2* consistently decreased, while *Wfs1* was decreased in CA1 excitatory neurons but increased in EC excitatory and inhibitory neurons. Taken together, these results suggest that enhancement of neurogenesis modulates the excitatory-inhibitory balance and expression profile within the hippocampal formation in the context of AD.

### 2.4 Glial profile in the hippocampal formation following enhanced neurogenesis in FAD

**Figure 4:**
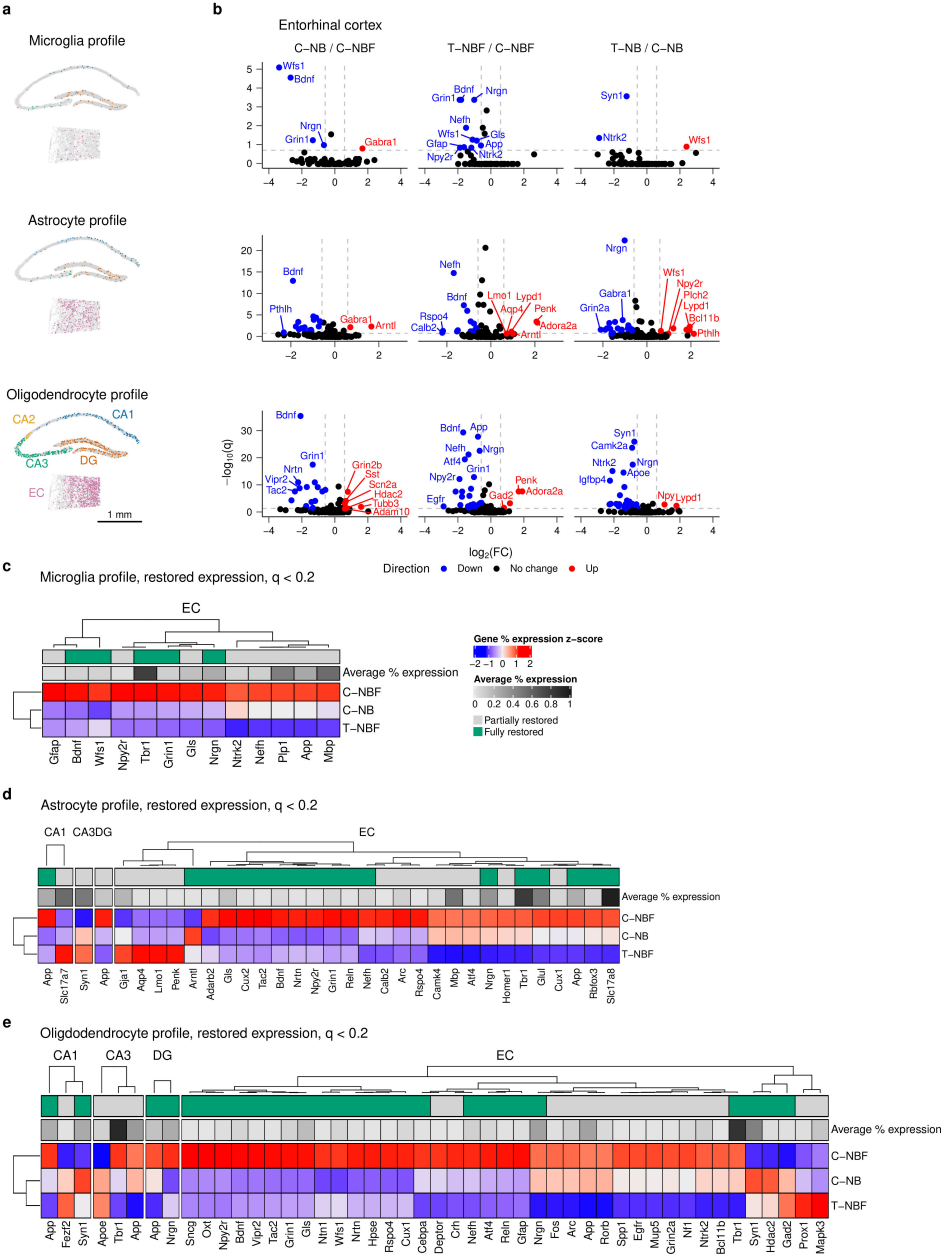
Glia differential gene expression analysis. **(a)** Representative scatter plot of glia cells from C–NB group showing location of microglia (top), astrocyte (middle), and oligodendrocyte profile cells (bottom) in the CA1, CA2, CA3, DG, and EC. Scale bar indicates 1 mm. **(b)** Volcano plots of differentially expressed genes in the EC for C–NB/C–NBF (left) and T–NBF/C–NBF (middle) and T–NB/C–NB (right) comparisons. **(c–e)** Heatmap of restored expression profile in each region for microglia **(c)**, astrocytes **(d)**, and oligodendrocytes **(e)**. Color indicates (column) *z*-scored percent expression of cells that were positive for that gene. Grayscale average percent expression bar indicates the average percent expression of that gene across all cells in each region. Green indicates genes that were fully restored.

We next asked whether altered neurogenesis would affect the profile of glia in the hippocampal formation. We first examined the spatial distribution of microglia, astrocytes, and oligodendrocytes (Figure 4a). We compared differentially expressed genes in microglia, astrocytes, and oligodendrocytes in each region of the hippocampal formation in C–NB/C–NBF and T–NBF/C–NBF (Figure 4b, Supplementary Figure S5). In microglia, we observed six differentially expressed genes for C–NB/C–NBF, ten differentially expressed genes for T–NBF/C–NBF, and five differentially expressed genes for T–NB/C–NB. For C–NB/C–NBF, *Grin2b* was upregulated in the DG and *Gabra1* was upregulated in the EC (Figure 4b, Supplementary Figure S5a). *Wfs1, Bdnf, Grin1*, and *Nrgn* were downregulated in the EC. For T–NBF/C–NBF, *Grin1, Npy2r, Bdnf, Gfap, Nefh, Ntrk2, Wfs1, Nrgn, Gls*, and *App* were all downregulated in the EC (Figure 4b). For T–NB/C–NB, *Wfs1* was upregulated in the EC, *Ntrk2* and *Syn1* were downregulated in the EC, and *Mapk3* and *Gfap* were downregulated in the DG. Thirteen genes were restored in microglia, all occurring within the EC and with the pattern being reduced expression in T–NBF relative to C–NBF: *App, Bdnf, Gfap, Gls, Grin1, Mbp, Nefh, Npy2r, Nrgn, Ntrk2, Plp1, Tbr1*, and *Wfs1* (Figure 4c).

In astrocytes, there were 23 differentially expressed genes in C–NB/C–NBF, 27 differentially expressed genes in T–NBF/C–NBF, and 34 differentially expressed genes in T–NB/C–NB, with the majority of these genes being located in the EC (Figure 4b, Supplementary Figure S5b). In C–NB/C–NBF, *Arntl* and *Gabra1* were upregulated, and the highest downregulated genes included *Bdnf, Vipr2, Adarb2, Wfs1*, and *Grin1*. For T–NBF/C–NBF, the most downregulated genes included *Calb2, Rspo4, Nefh, Gabra2*, and *Tac2*. The most upregulated genes included *Adora2a, Penk, Arntl, Lypd1*, and *Aqp4*, all in the EC. For T–NB/C–NB, the most downregulated genes included *Syn1* (CA1), *Ntrk2* (CA3), *Gfap* (CA3), *Nrgn* (EC), and *Gabra1* (EC). The most upregulated genes included *Pthlh, Npy2r, Wfs1, Bcl11b*, and *Tac1*. Thirty-one genes were significantly altered in the C–NBF group compared to C–NB and were fully or partly restored (Figure 4d). Like neurons, most of the restored genes in astrocytes were located in the EC, with the most common profile being downregulated genes in the T–NBF group relative to C–NBF. Among these genes, the most highly expressed included *Slc17a8, Tbr1, Slc17a7, Mbp*, and *Syn1*.

In oligodendrocytes, we observed 30 differentially expressed genes in C–NB/C–NBF, 33 differentially expressed genes in T–NBF/C–NBF, and 59 differentially expressed genes in T–NB/C–NB, the majority of which were in the EC and were downregulated in T–NB. For C–NB/C–NBF, the most significant upregulated genes included *Gad2* (CA3), *Grin2b* (EC), *Syn1* (CA1), *Sst* (EC), and *Scn2a* (EC), and the most downregulated genes were located in the EC and included *Nrtn, Bdnf, Npy2r, Grin1*, and *Wfs1*. For T–NBF/C–NBF, the most significant upregulated genes were *Gad2, Penk*, and *Adora2a* and the most downregulated genes were *Unc* For T–NB/C–NB, the most significant of these included *Syn1, Camk2a, Nrgn, Ntrk2*, and *Apoe* that were downregulated and *Npy* (EC), *Ngrn* (DG), *Npy* (EC), *Lypd1* (EC) and *Map2* (DG) were upregulated. We examined genes in the restored expression profile (*q* < 0.2) for oligodendrocytes, as described above, and found that there were 41 unique restored genes, with the majority found in the EC (Figure 4e). The most highly expressed restored genes included *Tbr1, App, Nrgn, Gls, Arc, Ntrk2, Nefh, Grin1, Bdnf* , and *Wfs1* (Figure 4e).

### 2.5 Layer differential gene expression and spatial profile of the entorhinal cortex

**Figure 5:**
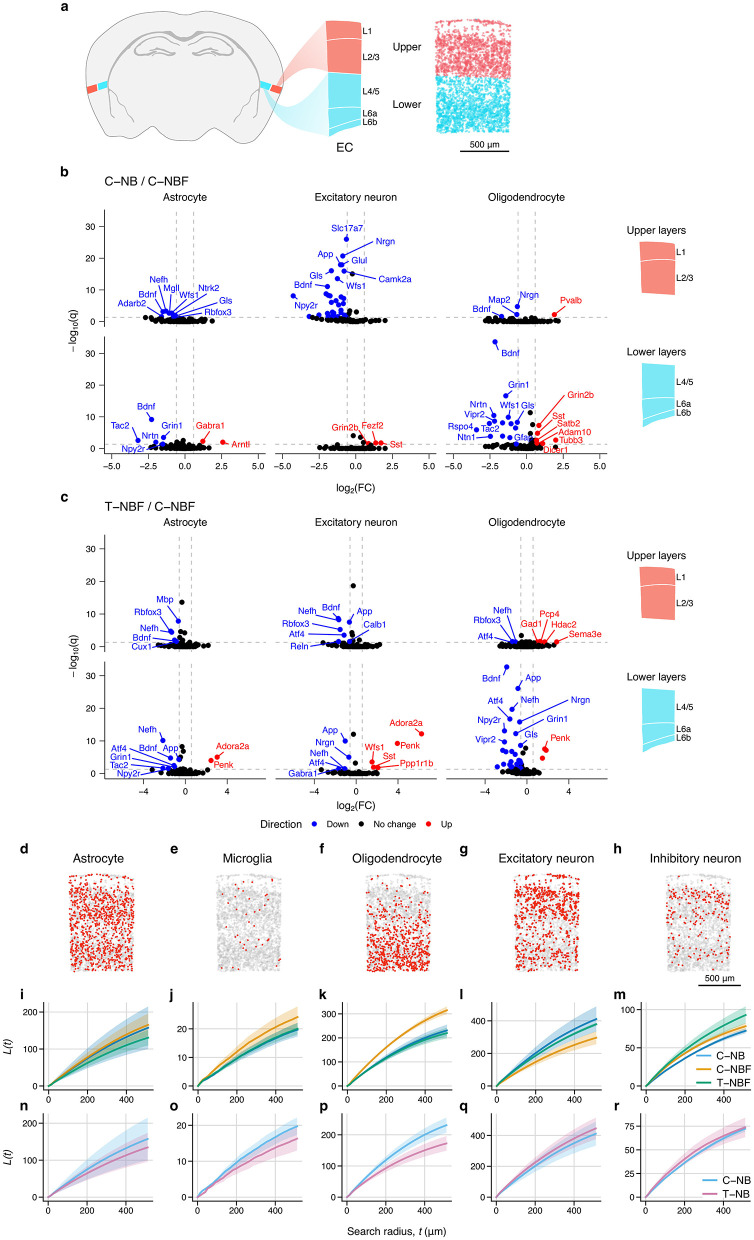
Entorhinal cortex layer and spatial statistics. **(a)** Atlas schematic of entorhinal cortex and location and layers. Diagram on the right shows upper (1–3) and lower (4–6) layers of the EC highlighted in red and blue, respectively. Representative scatter plot depicts estimated layers based on layer-specific marker expression used for differential gene expression analysis. **(b, c)** Volcano plots of differentially expressed genes in the upper and lower-layers of the EC for C–NB/C–NBF **(b)** and T–NBF/C–NBF **(c)**. **(d–i)** Spatial distribution of cell types in EC. Representative scatter plot of astrocytes **(d)**, microglia **(e)**, oligodendrocytes **(f)**, excitatory neurons **(g)**, and inhibitory neurons **(h)** within the EC. Scale bar indicates 500 μm. **(i–r)**: Average Ripley's *L* statistic for each cell type for each group for C–NB, C–NBF, and T–NBF **(i–m)** and C–NB and T–NB **(n–r)**. Line represents mean. Shaded area represents SEM. Blue: C–NB; pink: T–NB; orange: C–NBF; green: T–NBF.

Across both neurons and glia, we observed that the EC had the largest number of differentially expressed genes compared to other regions. To better characterize these changes in the EC, we examined the spatial distribution of gene expression within each cell type. We approximated the boundaries of the upper (1–3) and lower (4–6) layers of the EC (Figure 5a), and performed differential gene expression analysis between groups (Figures 5b, c). There were many layer- and cell-type specific changes in gene expression, with the largest number of changes occurring in upper-layer excitatory neurons and lower-layer oligodendrocytes. Upper-layer excitatory neurons had 36 genes that were upregulated in C–NBF relative to C–NB, including *Slc17a7, App, Nrgrn,a Glul, Gls, Camk2a, Wfs1, Npy2r*, and *Bdnf* . Notably, *Bdnf* was upregulated in C–NBF for almost all cell types and layers. Conversely, oligodendrocytes in the lower layers of the EC had many more differentially expressed genes. In C–NBF, *Grin2b, Sst, Satb2, Adam10, Tubb3*, and *Dicer1* were found to be downregulated relative to C–NB, while *Bdnf* was the most significant upregulated. In T–NBF/C–NBF, *App* was found to be reduced in T–NBF in both upper- and lower-layer excitatory neurons and lower-layer astrocytes and oligodendrocytes (Figure 5c). Together, this suggests that there were layer-specific differences in cell type expression profiles between groups in the EC.

In addition to changes in the gene expression profile, we tested if there were changes in the spatial distribution of cell types across groups (Figures 5d–r). We measured Ripley's *L* statistic (Ripley, 1976; Palla et al., 2022) for each cell type, which measures the spatial clustering for each cell type within the EC. Representative scatter plots of the EC show the spatial distribution for each cell type (Figures 5d–h). While the distribution of astrocytes was similar across the groups (Figures 5i, n), there was slight increase in clustering of microglia (Figure 5j) and oligodendrocytes (Figure 5k) in C–NBF, whereas the C–NB and T–NBF showed comparable values. Conversely, for excitatory neurons, C–NBF showed the lowest *L* statistic relative to C–NB and C–NBF (Figure 5m), while T–NBF showed the highest *L* statistic for inhibitory neurons relative to the other groups. In comparing the T–NB and C–NB groups (Figures 5n–r), the C–NB group showed greater clustering of microglia and oligodendrocytes in C–NB compared to T–NB, while astrocytes, excitatory neurons, and inhibitory neurons appeared comparable. Together, this data suggests that, in addition to changes in gene expression profile, the spatial distribution and clustering of cell types within the EC is possibly altered by both AD pathology and enhanced survival of newborn neurons.

## 3 Discussion

Mapping the transcription profile of the cellular constituents of the hippocampal formation in wild-type and FAD mice with varying levels of hippocampal neurogenesis provides several intriguing insights. First, it shows that the profile of neurons and glia in the hippocampus is modulated by levels of neurogenesis in the dentate gyrus. We show that these changes are not confined only to neurons that form synapses with new neurons but take place all over the hippocampal formation, especially in the DG, CA1, and the EC (Figure 5). Given that hippocampal neurogenesis is responsive to numerous conditions, e.g., environmental factors, aging, disease, our observations imply that these conditions may directly and indirectly affect the entirety of the hippocampal formation.

Previous studies have shown that increased adult neurogenesis is associated with changes in DG volume (Kempermann et al., 1997). Here, we did not observe any statistically significant differences in the area of the DG in our sections. However, this could simply be a limitation of extrapolating from a small number of 10 μm slices for spatial transcriptomics analysis and inherent variability in the slice preparation and selection. Further stereological investigation using 3D spatial transcriptomics with multiple slices of the DG would be necessary to fully understand the relationship between cell density and volume as it relates to the transcription profile of adult neurogenesis.

Previous studies using single-cell sequencing have provided detailed analyses of cell type differential gene expression in AD (reviewed in detail in Murdock and Tsai (2023)). However, the lack of spatial specificity has made it difficult to assess the specific differences in transcriptome profile between regions. Recently, the development of spatial transcriptomics technologies has begun to address this challenge (Moses and Pachter, 2022). Our study provides novel information on the neurogenesis-dependent spatial transcriptome profile in wild-type and AD. Recently, we have shown that augmentation of neurogenesis rescues memory performance in FAD mice by recruiting adult-born neurons (ABNs) to the engram, rescues dendritic spine density in immature and mature neurons, and rescues the transcription profile of engram neurons in the DG (Mishra et al., 2022). In particular, adult-born neurons that participated in the engram showed differential expression of key genes implicated in AD and neurogenesis such as *Bdnf*, *App, Adam10*, and *Apoe* (Mishra et al., 2022). In our previous study, we focused on the spatial transcription alterations just within the DG between engram and nonengram neurons. Here, we have widened our scope to the broader hippocampal circuit and across neurons and glia. To our knowledge, the current study is the first to analyze how the level of neurogenesis alters the spatial transcriptome profile in AD.

Second, enhancing neurogenesis in wild-type and FAD mice has distinct effects. The 5XFAD mice used in this study develop progressive pathology, manifested by a different profile of neurons and glia compared to age- and gender-matched wild-type mice. The 5XFAD transgenic mouse model of FAD harbors five FAD mutations (three in *App* and two in *Ps1*) that elicit a rapid and robust FAD-like phenotype. Intraneuronal A_42_ can be found at approximately 1.5 months of age followed by plaque deposition at approximately two months of age. Further, synaptic loss and neurodegeneration have been characterized starting at approximately four months of age, with a dramatic decline by nine months of age (coupled with neuronal loss starting at this age). Behaviorally, these mice develop deficits in spatial memory by four months of age (Oakley et al., 2006). These mice show deficits in adult neurogenesis around two months of age, which roughly coincides with the onset of plaque deposition (Moon et al., 2014; Mishra et al., 2022). Interestingly, enhancing neurogenesis in these mice (T–NBF) altered neuronal transcription to exhibit a neuronal profile that resembled the profile of the wild-type (C–NB), rather than the FAD one (Figures 1n, Figures 2f–k). This suggests that some of the neuronal profile in the hippocampus in FAD can be attributed to deficits in neurogenesis. Transcription profile restoration in 5XFAD mice following enhanced neurogenesis was further observed in the analysis of excitatory and inhibitory neuronal profiles (Figure 3), as well as in microglia, oligodendrocytes, and astrocytes (Figure 4).

Third, we observed that, while the number of neurons was comparable in the hippocampal formation in all groups, an attempt to sub-classify neurons into excitatory and inhibitory types revealed significant shifts in the profile of neurons, clearly revealing alterations in the transcription of inhibitory and excitatory signals (Figure 3). These results may provide a molecular profile underlying recent functional evidence of excitatory-inhibitory imbalance in AD (Najm et al., 2019; Toniolo et al., 2020; Arroyo-García et al., 2021; Lauterborn et al., 2021; Morrissey et al., 2023; Fortel et al., 2020). Further, the transcription of other signals that are not implicated in excitation or inhibition was altered in these neurons following augmentation of neurogenesis. This may suggest that levels of neurogenesis affect the extent of inhibitory or excitatory tone of neurons in the hippocampus by regulating signal transcription. Interestingly, enhancing neurogenesis in wild-type and in FAD led to opposite effects compared to their respective baselines. That said, we did observe expression of excitatory markers in inhibitory neurons and vice-versa (Figures 3f–h), which may suggest that individual marker expression is not sufficient for identifying *bona fide* excitatory/inhibitory populations. Further experiments will be needed to conclusively determine the co-expression of putative cell type markers.

Fourth, levels of neurogenesis, in both wild-type and FAD, modulated the transcription profile of glia in the hippocampus, and most importantly, primarily in the EC (Figure 1n). Previous studies have demonstrated that glia play an important role in AD pathology (Murdock and Tsai, 2023; Sadick et al., 2022; Bartzokis, 2011) and in modulating hippocampal neurogenesis (Gonçalves et al., 2016; Ashton et al., 2012). Notably, a single-cell sequencing study by Mathys et al. (2019) found that the top 5 % most DEGs in human AD prefrontal cortex samples were observed across multiple cell types, suggesting that AD affects the profile of a variety of cell types. Similarly, we found many recurring genes differentially expressed across cell types. They noted that many of the DEGs were related to oligodendrocyte and myelin pathways, of which, we found oligodendrocytes to have some of the largest number of DEGs among glia, along with differential expression of myelin-related genes including *Mbp* and *Plp1*.

Fifth, while the density of cells is largely similar, the spatial distribution of cells in the EC is modulated by the level of neurogenesis and FAD (Figures 5d–r). The entorhinal cortex plays a critical role in episodic, spatial, and semantic memory, and is one of the areas affected earliest in AD (Igarashi, 2023). Anatomically, the primary input to the DG occurs via the perforant pathway from neurons in EC layer II (ECx-II). The EC also projects to the CA1 and CA3, primarily via ECx-III, while output from the hippocampus projects back to ECx-IV. The EC itself receives input from parts of the cortex. Thus, the EC has extensive connectivity with the hippocampus and facilitates its communication with the cortex (Igarashi, 2023; Fyhn et al., 2004; Van Groen et al., 2003; Suh et al., 2011).

The EC is known to modulate neurogenesis (Gama Sosa et al., 2004). Furthermore, deep brain stimulation of the EC is known to rescue memory performance in FAD mouse models and requires neurogenesis, which is mediated in part by insulin signaling (Stone et al., 2011; Ronaghi et al., 2019; Chavoshinezhad et al., 2021). ECx-II is particularly vulnerable in AD, as well as ECx-IV. Thus, there is a critical relationship between the EC, neurogenesis, and memory performance in AD. To our knowledge, our results are the first to implicate the reciprocal modulation of enhanced hippocampal neurogenesis on the EC in wild-type and FAD mice. Future work examining the functional implications of these changes will shed more light on how neurogenesis rescues memory performance and the transcriptomic profile in the EC in AD.

Beyond an effect on the hippocampal formation, the extent of hippocampal neurogenesis, the function of its cellular constituents, and the composition of the hippocampal neurogenic niche may affect the homeostasis and function of other brain areas, including the other neurogenic niche in rodents, i.e., the subventricular zone and the cerebrospinal fluid (Salta et al., 2023; Disouky and Lazarov, 2021). This has major implications for AD, where many of the players of this complex interplay of cell types and signaling pathways involved in the neurogenic niche are impaired in AD (see review by Salta et al., 2023 for details). This may include the loss of neurotrophic characteristics, effect on endothelial cell structure and function (Fainstein et al., 2018), as well as the regulation of other cell types and homeostasis, for example, microglia (Lachish et al., 2022). Lugert et al. (2017) found that Gpc2, secreted by immature neurons, is involved in regulating Fgf-mediated NPC proliferation and is also detected in CSF. The CSF is known to have factors that are critical for growth and survival of NPCs (Lehtinen and Walsh, 2011). Thus enhanced neurogenesis may also impact the composition of the CSF as well. Together, this highlights that alterations in the level of adult neurogenesis affects not only the number of neurogenic cells and alters their genetic profile, but also likely has wider effects on the composition and maintenance of the neurogenic niches and CSF.

Our analyses were performed specifically in the anterior portion of the hippocampus. However, the hippocampus has notable differences in function (Moser and Moser, 1998; Moser et al., 1993), cell type composition (Yamada and Jinno, 2014; Jinno and Kosaka, 2009, 2006; Jinno et al., 1998), and neurogenesis (Jinno, 2011a,b) along its longitudinal axis. Thus, it is possible that the spatial transcriptome profile we observed may be different along the longitudinal axis of the hippocampus. Future studies examining ventral portions of the hippocampus or 3D spatial transcriptomics techniques will provide better insight into the broader arrangement of spatial gene expression in the hippocampus. Additionally, while our sample size is comparable to other spatial transcriptomics studies (Chen et al., 2020; Bhattacherjee et al., 2023; Sun et al., 2024; Zeng et al., 2023; Habib et al., 2020), future experiments with larger sample size, age groups, and AD mouse models would determine how robust these findings are. While the brain sections for this study were processed in a way that aligns with the *in situ* sequencing, they are inadequate for immunohistochemistry, including very thin (10 μm) sections. Future experiments that investigate the role of the top targets identified here will be insightful. Finally, while we used individual cell type proxies to identify (mutually exclusive) putative cell types, more probabilistic cell type annotation techniques (Chen et al., 2020; Zhou et al., 2024; Qian et al., 2020) or tissue domain analyses (Hu et al., 2021; Shang and Zhou, 2022) may provide more nuance to our findings and would be an interesting foundation for future work. In summary, we provide a detailed resource and characterization of the spatial genetic profile of a multiple cell types in the hippocampal formation in both healthy and FAD mice with varying levels of neurogenesis. Together, these data demonstrate useful insights into how neurogenesis and FAD influence the spatial genetic landscape of the hippocampal formation and can provide a foundation for future work examining the molecular mechanisms underlying these processes.

## 4 Methods

### 4.1 Animals

Animal experiments were approved by and carried out in accordance with the Institutional Animal Care and Use Committee at the University of Illinois Chicago (ACC Protocol #20–123; PI: Lazarov) and reported in accordance with the ARRIVE guidelines (Percie Du Sert et al., 2020). Mice were housed in standard housing cages under a 12 h light-dark cycle and allowed to eat and drink *ad libitum*. Mice used in this study were from a C57Bl/6 background and generated from *Nestin-CreER*^*T2*^ mice crossed with *Bax*^*fl/fl*^ mice to obtain *Nestin-CreER*^*T2*^*;Bax*^*fl/fl*^ (NB) mice, courtesy of Dr. René Hen (Columbia University, New York, NY). The NB mice were crossed with 5XFAD mice (The Jackson Laboratory, catalog #034848) to generate *Nestin-CreER*^*T2*^*;Bax*^*fl/fl*^*;5XFAD* (NBF) mice. As described in Mishra et al. (2022), deletion of *Bax* was induced by intraperitoneal injection of 2 mg of tamoxifen (Sigma-Aldrich, T–5648) dissolved in 20 mg/mL in corn oil once per day for 5 d at 4 w of age. The NB and NBF mice that received only corn oil as a vehicle control are denoted C–NB and C–NBF, respectively, and the NB and NBF mice that received tamoxifen are denoted T–NB and T–NBF, respectively. Animals were put on a 40 mg/kg doxycycline-supplemented diet 1 d before surgery. The engram viral cocktail (AAV9–c-fos–tTA and AAV9–TRE–ChR2–eYFP) was injected bilaterally at four months of age. Two weeks later mice underwent contextual fear conditioning where they were received a foot shock in the chamber on the first day and placed in the chamber the next day to measure freezing behavior (full behavioral experiment details described in Mishra et al., 2022). Animals were kept on doxycycline-supplemented diet until the first day of contextual fear conditioning and returned to doxycycline-supplemented diet after the training. Mice were sacrificed at 4.5 months of age. Two female mice from each group (C–NB, T–NB, C–NBF, and T–NBF) were used in this study, with a total of three samples for each group (i.e., two samples were obtained from one animal from each group). One investigator randomly assigned mice to corn and tamoxifen treatment groups. Another investigator blinded to condition performed tasks (e.g., behavior, perfusion, and tissue preparation).

### 4.2 Immunofluorescence staining

Animals were transcardially perfused with PBS followed by a 4 % w/v paraformaldehyde (PFA) solution. Brains were removed and kept in 4 % w/v PFA to fix for 24 h at 4 °C. Brains were treated with 15 % and 30 % sucrose, and 30 μm thick coronal sections were cut and collected in cryoprotectant solution. For immunofluorescence staining, sections were collected in a 24-well plate, washed with PBS three times for 5 min each, and incubated with blocking solution (tris-buffered saline (TBS) with 0.25 % v/v Triton X–100, 5 % v/v normal donkey serum) for 1 h, followed by incubation with primary antibodies for 48 h. After incubation with primary antibodies, sections were washed with TBS three times, followed by incubation with secondary antibodies for 3 h at room temperature. Sections were washed with TBS three times and counterstained with 1:10.000 DAPI (1 μg/mL) for 10 min, followed by two TBS washes. Sections were mounted on a glass slide covered with fluorescence antifade mounting media and coverslips. Imaging was performed on a Zeiss LSM 710 confocal microscope. Primary antibodies: 1:400 rabbit anti-doublecortin (Cell Signaling #4604, RRID: AB_561007), 1:500 mouse anti-NeuN (Abcam ab104224, RRID: AB_10711040). Secondary antibodies: 1:500 donkey anti-rabbit Cy3 (Jackson ImmunoResearch RRID: AB_2307443), 1:500 donkey anti-mouse Cy5 (Jackson ImmunoResearch RRID: AB_2340820) and DAPI (Invitrogen, D1306).

### 4.3 Brain tissue isolation and *in situ* sequencing for spatial transcriptomics

Brain tissue collected for spatial transcriptomics analysis was performed as described in our previous study (Mishra et al., 2022). Briefly, mice were anesthetized with isoflurane and transcardially perfused with ice-cold RNAse-free phosphate-buffered saline (PBS; Invitrogen AM9625) for 2 min. Coronal cryosections of the brain were prepared at 10 μm thickness of the dorsal hippocampus (approximately -2 mm from bregma). Sections were maintained on dry ice and processed by Cartana (Sweden, now 10X Genomics) for spatial transcriptomics. Full details of the spatial transcriptomics processing pipeline is described in detail in Mishra et al. (2022). Briefly, brains were harvested following transcardial perfusion of RNA-freeze PBS solution and then flash-frozen in Tissue-Tek solution. Brain sections were prepared at 10 μm thickness according to Cartana's sample preparation instructions and were shipped overnight to Cartana (now 10X Genomics) for processing. Samples were fixed, permeabilized, and chimeric padlock probes containing barcodes specific for the pre-defined panel of 159 genes of interest were added and allowed to hybridize and amplify via rolling circle amplification process overnight. After quality control, 6 different flourophores were added to image each gene-specific barcode. The raw images files and CSV files containing the decoded gene labels, and image coordinates were saved as raw data for downstream processing. All steps were strictly adhered to Cartana's instructions without deviation.

### 4.4 Gene panel

There were 158 genes of interest probed for *in situ* sequencing analysis in the mouse tissue, as described in our previous study (Mishra et al., 2022): *Ache, Acta2, Adam10, Adamts2, Adarb2, Adora2a, Aldoc, Apoe, App, Aqp4, Arc, Arhgap36, Arntl, Atf4, Atxn1, Bace1, Bcl11b, Bdnf, Calb1, Calb2, Calca, Camk2a, Camk4, Cav1, Cblb, Cbln2, Cck, Ccr5, Cebpa, Chat, Chodl, Chrna2, Chrna6, Cnmd, Cpa6, Creb1, Crh, Crhr1, Crhr2, Crispld2, Cux1, Cux2, Dcn, Dcx, Deptor, Dicer1, Drd1, Egfr, Egr1, Eif2ak4, Eif2s1, Fev, Fezf2, Fos, Foxp2, Gabra1, Gabra2, Gad1, Gad2, Gfap, Gja1, Gls, Glul, Grin1, Grin2a, Grin2b, Hdac2, Homer1, Hpse, Igfbp4, Itgam, Kcnj8, Krt73, Lamp5, Laptm5, Lhx6, Lmo1, Lypd1, Map2, Mapk3, Mapt, Mbp, Mgll, Mmp9, Mup5, Ncam1, Ndnf, Nefh, Nes, Neurod1, Neurod6, Nf1, Npas4, Npy, Npy2r, Nrgn, Nrtn, Nt5c1a, Ntn1, Ntrk2, Nts, Oprk1, Oxt, P2rx3, Pcp4, Pdyn, Penk, Per1, Plat, Plch2, Plcxd2, Plp1, Ppp1r1b, Prox1, Psen1, Pthlh, Ptn, Ptprc, Pvalb, Rbbp4, Rbfox3, Reln, Rorb, Rprm, Rspo4, S1pr1, Satb2, Scn2a, Sema3e, Slc17a6, Slc17a7, Slc17a8, Slc6a1, Slc6a3, Slc6a4, Slc6a5, Sncg, Spp1, Sst, Syn1, Syt6, Sytl1, Tac1, Tac2, Tafa1, Tbr1, Th, Tpbg, Tph1, Trem2, Trh, Trhr, Trpv1, Tubb3, Unc5c, Vip, Vipr2*, and *Wfs1*.

### 4.5 Cell segmentation

Cell segmentation was performed on the DAPI images for each sample to assign gene spots to individual cells. Cell segmentation was performed using a custom MATLAB script (courtesy of Xiaoyan Qian, Cartana) as described previously (Mishra et al., 2022). The output of the cell segmentation is a gene × cell matrix ***X***∈ℝ^*m*×*n*^ for *m* genes and *n* cells for each image, where *x*_*ij*_ is the number of spots of the *i*th gene in the *j*th cell.

### 4.6 Region segmentation

Region masks of the brain were demarcated manually using the DAPI images in FIJI (Schindelin et al., 2012) for the dentate gyrus (DG), subgranular layer (SGL) of the DG, *cornu Ammonis* (CA) fields 1–3, and the entorhinal cortex (EC). The SGL was defined as the region just below the granule cell (GC) layer to approximately 1/3 of the height of the GC layer to account for the migration and maturation of adult-born neurons. The polygonal selection tool in FIJI was used to outline a mask for each region, and the (*x, y*)-coordinates of the polygon were then saved. Regions were annotated by consensus of at least two experimenters for each contour. Then, cells were assigned to each region if they were located within the convex hull described by the (*x, y*)-coordinates of the respective region masks using the matplotlib.path.Path.contains_points function from the matplotlib library (Hunter, 2007). The gene × cell matrices for each region were concatenated together to form a single gene × cell data frame with cell ID, subject ID, sample ID, region, and hemisphere label columns.

### 4.7 Cell type classification

Cell types were defined in a divisive hierarchical manner using *a priori* biological markers such that cell types were assigned to non-overlapping (sub)sets. Cells were defined as “neurons” if they were negative for all of: *Acta2, Aldoc, Aqp4, Dcn, Gfap, Gja1, Itgam, Kcnj8, Laptm5, Mbp, Plp1, and S1pr1*. The remainder of cells were defined as “nonneurons.” Neurons were defined as “immature neurons” if they were located in the sub-granular layer (SGL) of the DG and positive for any of: *Dcx, Ncam, Neurod1*, or if they were positive for *Prox1* and negative for *Rbfox3*. Neurons were defined as “mature neurons” if they were neurons not labeled as “immature.” Mature neurons were defined as “inhibitory” if they expressed any of *Map2, Prox1, Rbfox3, Syn1, Tubb3*, and expressed any of the inhibitory markers: *Adarb2, Arhgap36, Calb1, Calb2, Cck, Chodl, Chrna2, Cnmd, Crh, Crhr2, Crispld2, Gabra1, Gabra2, Gad1, Gad2, Hpse, Igfbp4, Krt73, Lamp5, Lhx6, Lmo1, Npy, Nrtn, Nts, Plch2, Pthlh, Pvalb, Rspo4, Sema3e, Sncg, Sst, Tac1, Tac2, Tafa1, Tpbg, Vip*, or *Vipr2*. The remainder of mature neurons that were not defined as inhibitory were defined as “excitatory.” For nonneurons, cells were defined as “astrocytes” if they expressed *Aldoc* and were negative for all of: *Laptm5, Acta2, Kcnj8*. Nonneurons were defined as microglia if they expressed *Laptm5* and were negative for all of: *Aldoc, Acta2, Kcnj8*. Nonneurons were defined as oligodendrocytes if they expressed *Mbp* and were negative for all of: *Acta2, Aldoc, Kcnj8, Laptm5*. Remaining nonneurons that did not meet these criteria were classified as “other.”

### 4.8 Statistical analysis

The cell density for a given cell type and region combination was calculated as the number of cells of that type divided by the area of the region measured using FIJI. One-way ANOVA was used to compare densities across groups. Differential gene expression was performed in a pairwise manner between groups by cell type and region. For a given cell type and region, Fisher's exact test (FET) was used to compare the counts of cells positive for a given gene in each group. Genes were defined as “restored” in the T–NBF group if the gene was statistically significantly differentially expressed in the T–NBF/C–NBF comparison and the sign of the fold change was the same in the C–NB/C–NBF comparisons, that is, the gene was up- or downregulated in the same way as the C–NB group. Genes were further defined as “fully” restored if the fold change between C–NB/C–NBF was also statistically significant. To compare restored genes in heatmaps, the percent expression of each gene was transformed to a *z*-score. Ripley's *L* statistic (Ripley, 1976; Palla et al., 2022) was used to compare the spatial clustering of cell types in the entorhinal cortex between groups.

#### 4.8.1 Differential gene expression

For each of the 158 genes, the contingency table for the *i*th gene was constructed as shown in Table 1. The odds ratio and *p* value were calculated for each gene. Benjamini-Hochberg False Discovery Rate (FDR) (Benjamini and Hochberg, 1995) was used to correct for multiple comparisons. The percent expression of each gene was used to calculate the log_2_(fold change) (FC) for pairwise comparisons. To avoid infinite values in the log_2_ transformation, the minimum percent expression value was added as a pseudovalue. Genes were considered up- or downregulated if FC > 1.5 or FC < 1/1.5.

**Table 1:** Example contingency table for the *i*th gene.

	**Group 1**	**Group 2**
Express gene_*i*_	*a*	*b*
Do not express gene_*i*_	*c*	*d*

The variables *a*, *b*, *c*, and *d* denote the number of cells for each category.

For the EC, the upper and lower-layer boundaries were estimated using *Calb1* expression as a proxy for the upper-layer. For each sample, the median *Calb1* (*x, y*)-coordinate was calculated. The upper- and lower-layer boundaries were estimated at one standard deviation from the median *Calb1* (*x, y*)-coordinate. Differential gene expression for each layer across groups was performed using FET as described above.

#### 4.8.2 Spatial statistics

To assess the spatial distribution of cells in the entorhinal cortex, we used Ripley's *K* function (Ripley, 1976; Palla et al., 2022), which measures the clustering/dispersion of points in space. Given *n* points with two-dimensional spatial coordinates $X=\left[x_{1},\ldots ,x_{n}\right]\in {\mathbb{R}}^{2\times n}$, where $x_{i}={\left[x_{1},x_{2}\right]}^{⊤}$ is the coordinate for the *i*th point, Ripley's *K* statistic is defined as


(1) $$\begin{array}{l} K\left(t\right)=\frac{1}{\text{λ}}\overset{n}{\underset{i=1}{\sum }}\overset{n}{\underset{\begin{array}{c} j=1\\ i\neq j \end{array}}{\sum }}\frac{I\left(d_{ij}<t\right)}{n}, \end{array}$$

where *d*_*ij*_ is the Euclidean distance between cells ***x***_*i*_ and ***x***_*j*_, *t* is the search radius, the indicator function *I*(·) = 1 if the operand is true and 0 otherwise, *n* is the number of cells, and λ is the average density, defined as λ = *n*/*A*, where *A* is the area of the region. We used the variance-stabilized form of Equation 1, known as Ripley's *L* statistic, which is defined as


$$\begin{array}{l} L\left(t\right)=\sqrt{\frac{K\left(t\right)}{\pi }}. \end{array}$$

Ripley's *L* statistic was computed for each cell type for each sample across a range of *t*. The mean *L*(*t*) and standard error of the mean (SEM) were computed for each group.

#### 4.8.3 Dimensionality reduction

To produce a low-dimensional embedding of the hippocampal formation, the rectangle bounded by the contour of the hippocampal formation was divided into equally spaced 50 μm2 bins, and the number of spots for each gene was calculated. The preprocessing pipeline from the squidpy (Palla et al., 2022) and scanpy (Wolf et al., 2018) python libraries were used that included median normalization (sc.pp.normalize_total function) and ln(1+*x*) transformation (sc.pp.log1p function) of the binned counts. Uniform manifold approximation and projection (UMAP) (McInnes et al., 2018) was used to reduce the data to two dimensions as implemented in the sc.tl.umap function.

### 4.9 Software

Cell segmentation was performed using MATLAB version 9.0.0.341360 R2016a (MathWorks, 2017). Analysis was performed using Python version 3.9.15 (Van Rossum and Drake, 1995) from the Anaconda distribution (Anaconda, 2018) and associated scientific computing libraries, including numpy (Harris et al., 2020), scipy (Virtanen et al., 2020), pandas (McKinney, 2010), scikit-learn (Pedregosa et al., 2011), and squidpy (Palla et al., 2022), and R version 3.6.3 (R Core Team, 2018). Visualization was performed using Python with matplotlib (Hunter, 2007) and seaborn (Waskom, 2021), and R with ggplot2 (Wickham, 2016) and ComplexHeatmap (Gu et al., 2016) packages. Arrangement of figures was done using Inkscape version 0.92 (Inkscape, 2017). The “mouse-darkgray” icon used in Figure 1a by Servier https://smart.servier.com/ is licensed under CC-BY 3.0 Unported https://creativecommons.org/licenses/by/3.0/. The “screen” and “workstation” icons used in Figure 1c were provided by https://bioicons.com/ by Simon Dürr and are licensed under CC0 https://creativecommons.org/publicdomain/zero/1.0/.

## Data Availability

The datasets presented in this study can be found in online repositories. The names of the repository/repositories and accession number(s) can be found below: https://doi.org/10.5281/zenodo.12810114.
