# Supplementary figures and images for: Neurogenesis drives hippocampal formation-wide spatial transcription alterations in health and Alzheimer's disease
Source: Front Dement. 2025 Apr 16;4:1546433. doi: 10.3389/frdem.2025.1546433 (PMC12041076; doi:10.3389/frdem.2025.1546433)

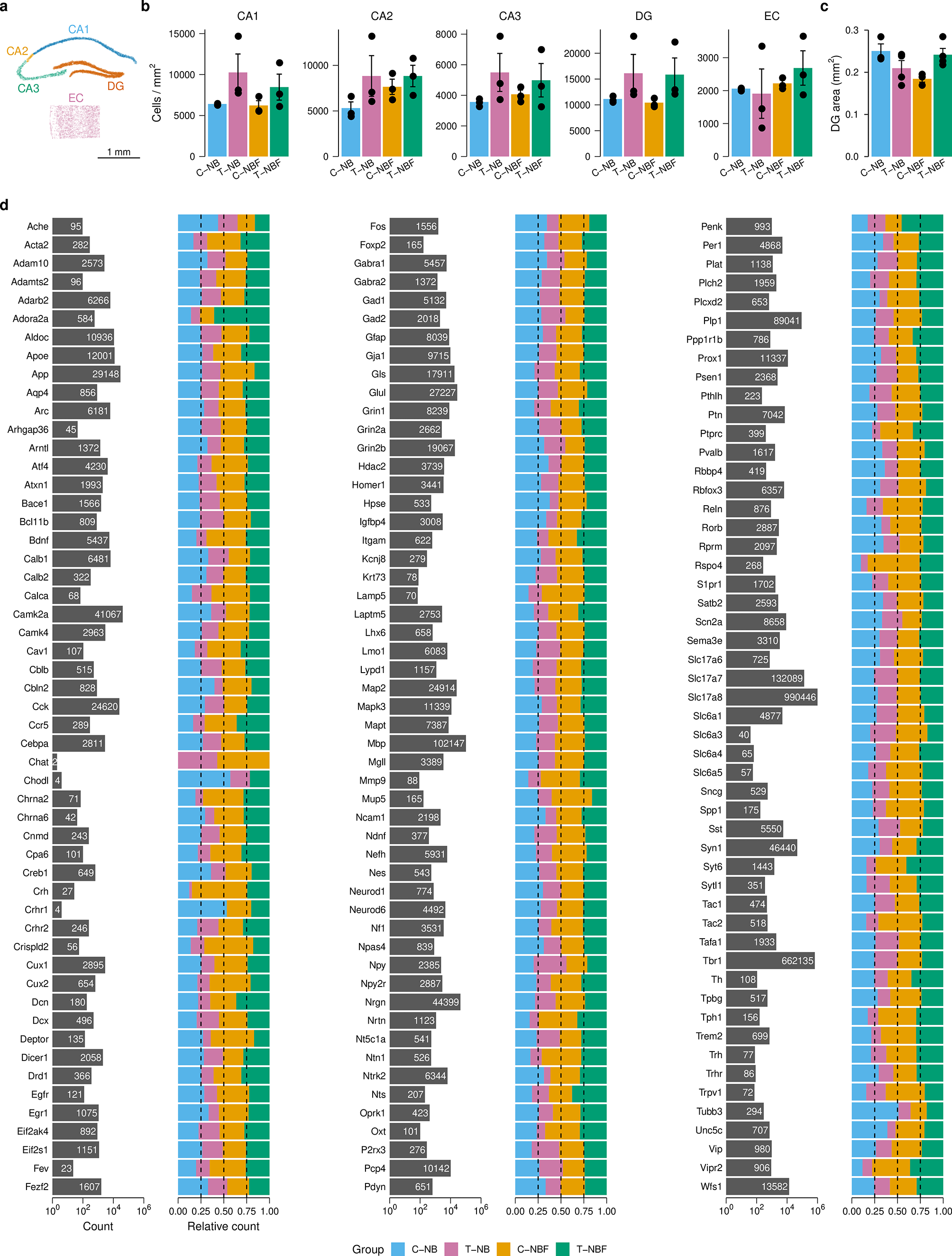

Supplement: Supplementary file 5 [file Image_1.tif]

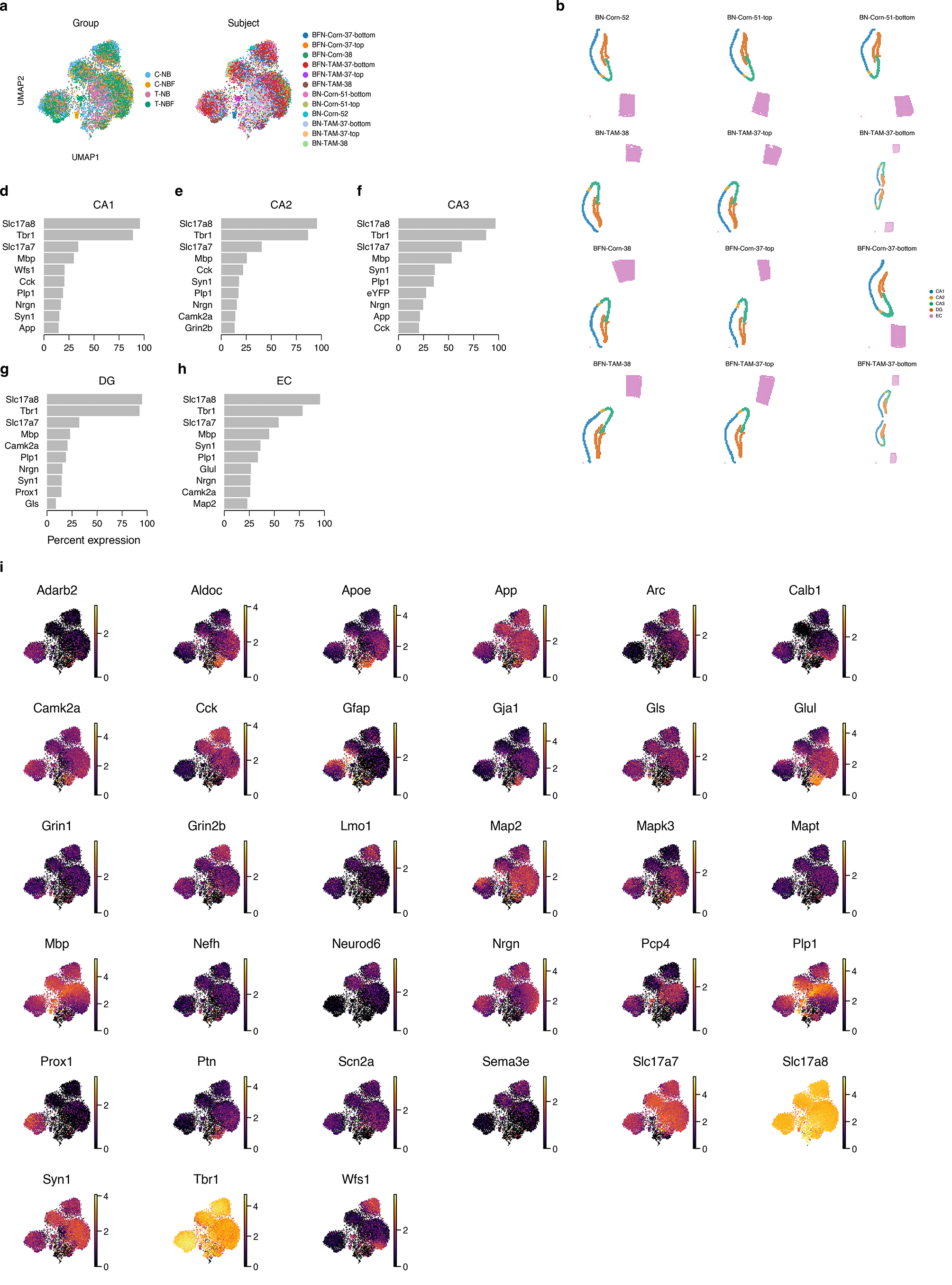

Supplement: Supplementary file 6 [file Image_2.tif]

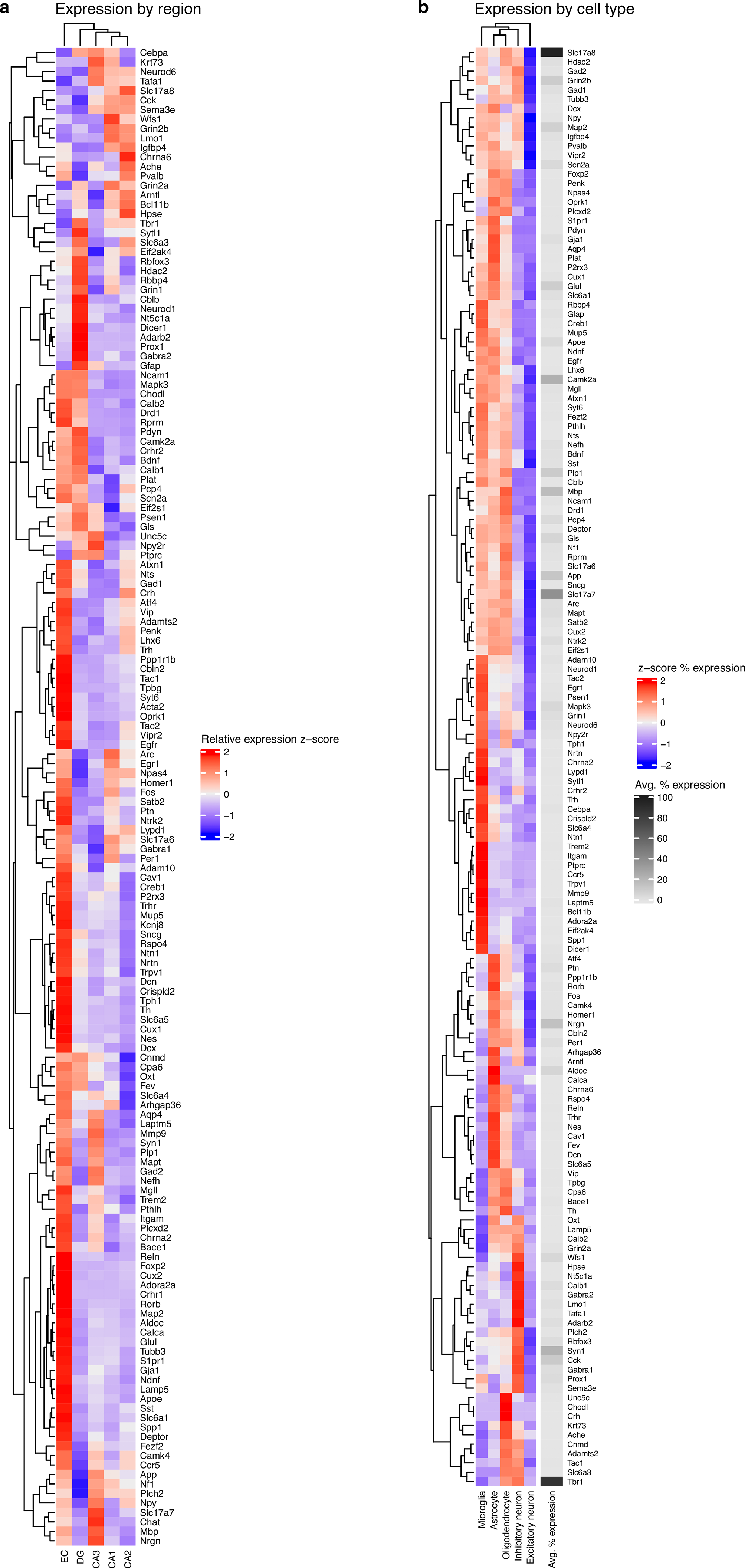

Supplement: Supplementary file 7 [file Image_3.tif]

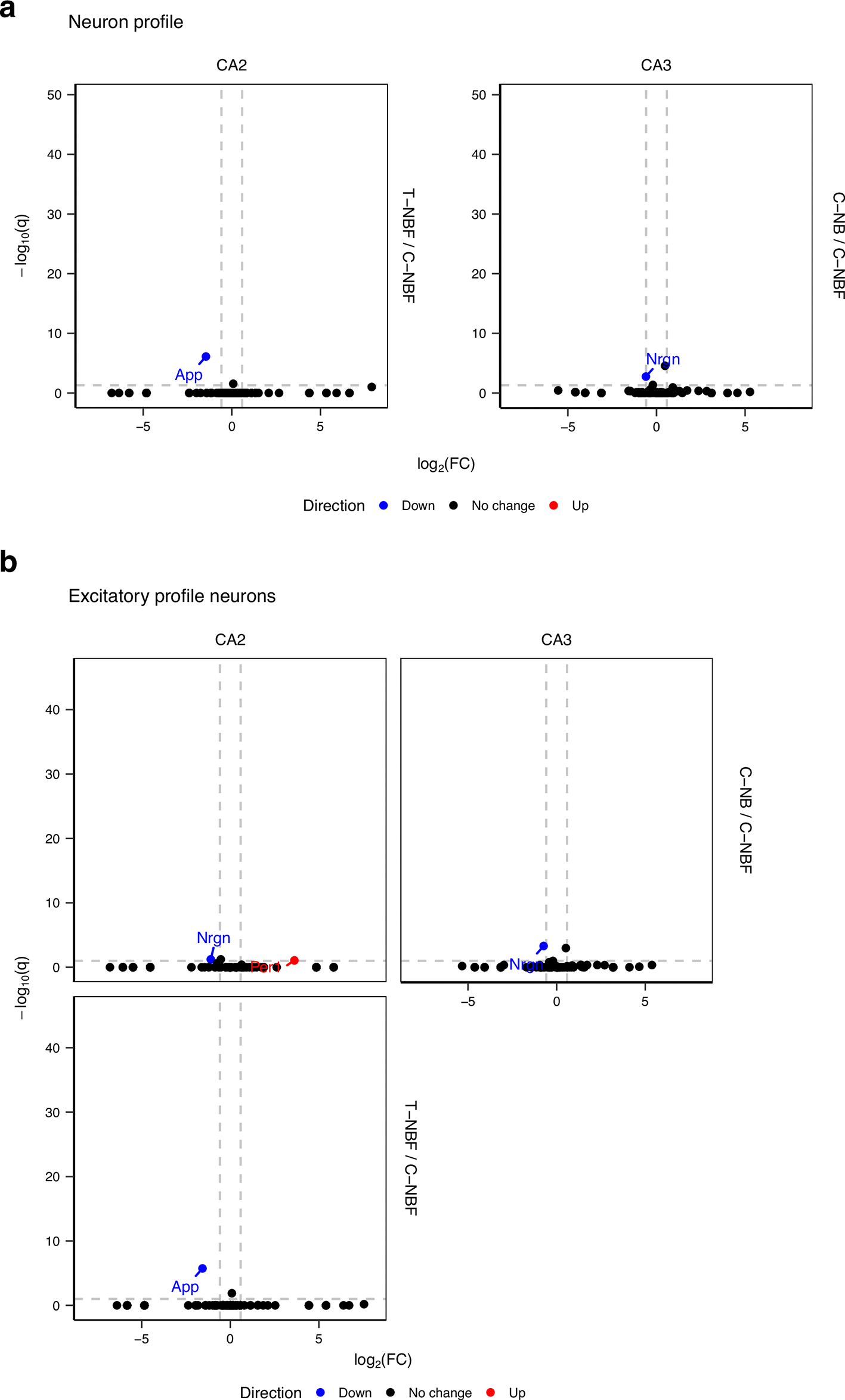

Supplement: Supplementary file 8 [file Image_4.tif]

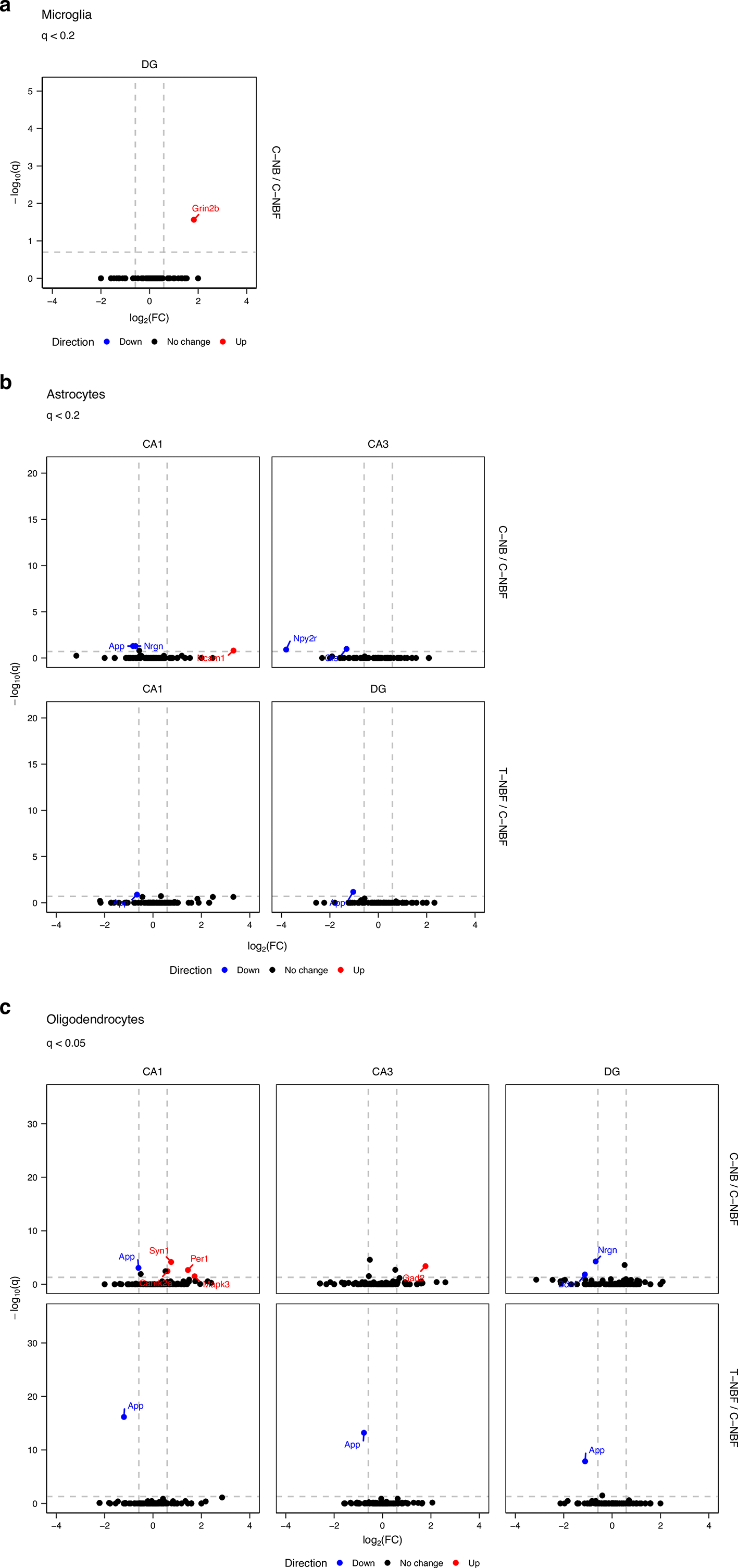

Supplement: Supplementary file 9 [file Image_5.tif]

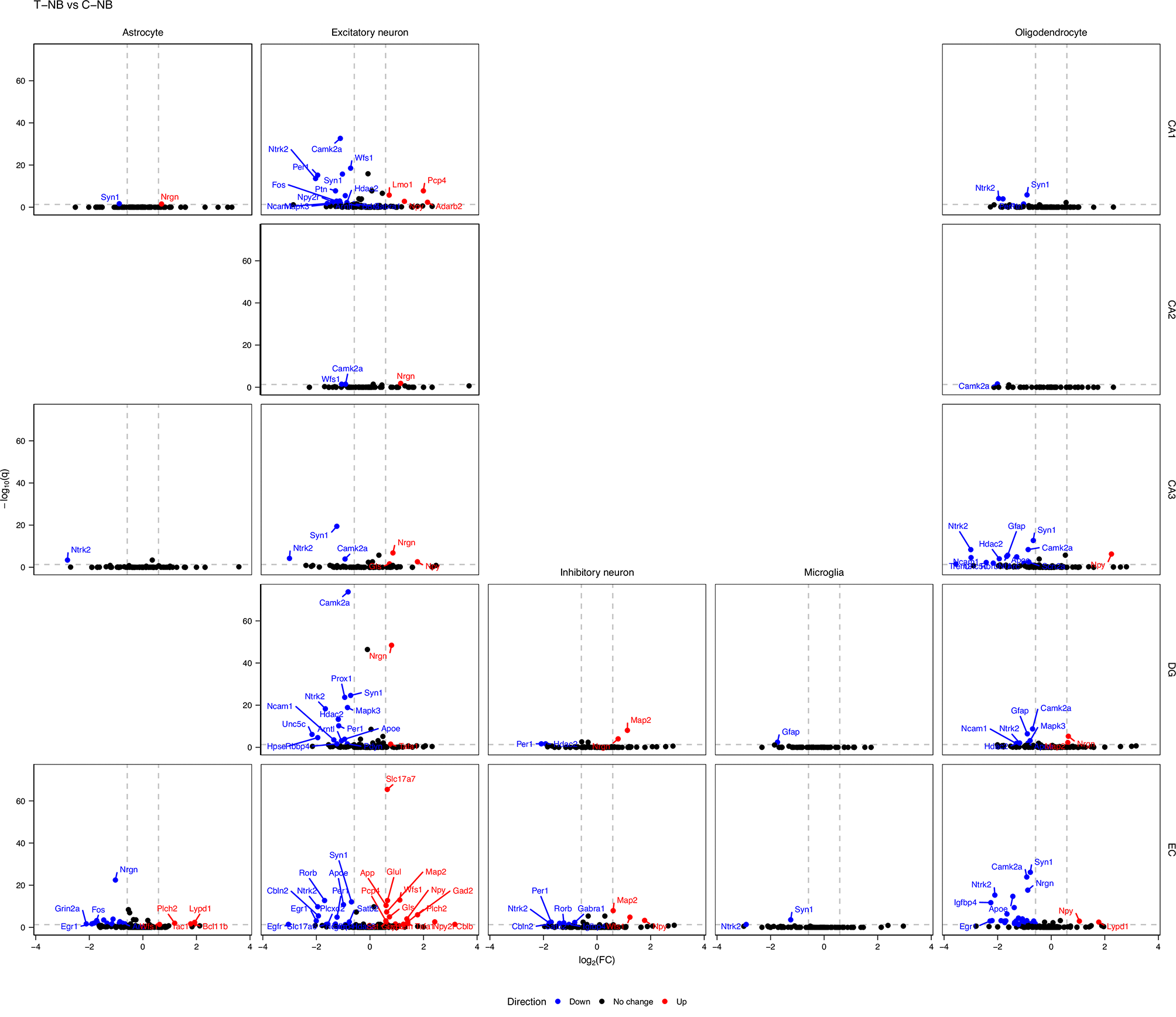

Supplement: Supplementary file 10 [file Image_6.tif]

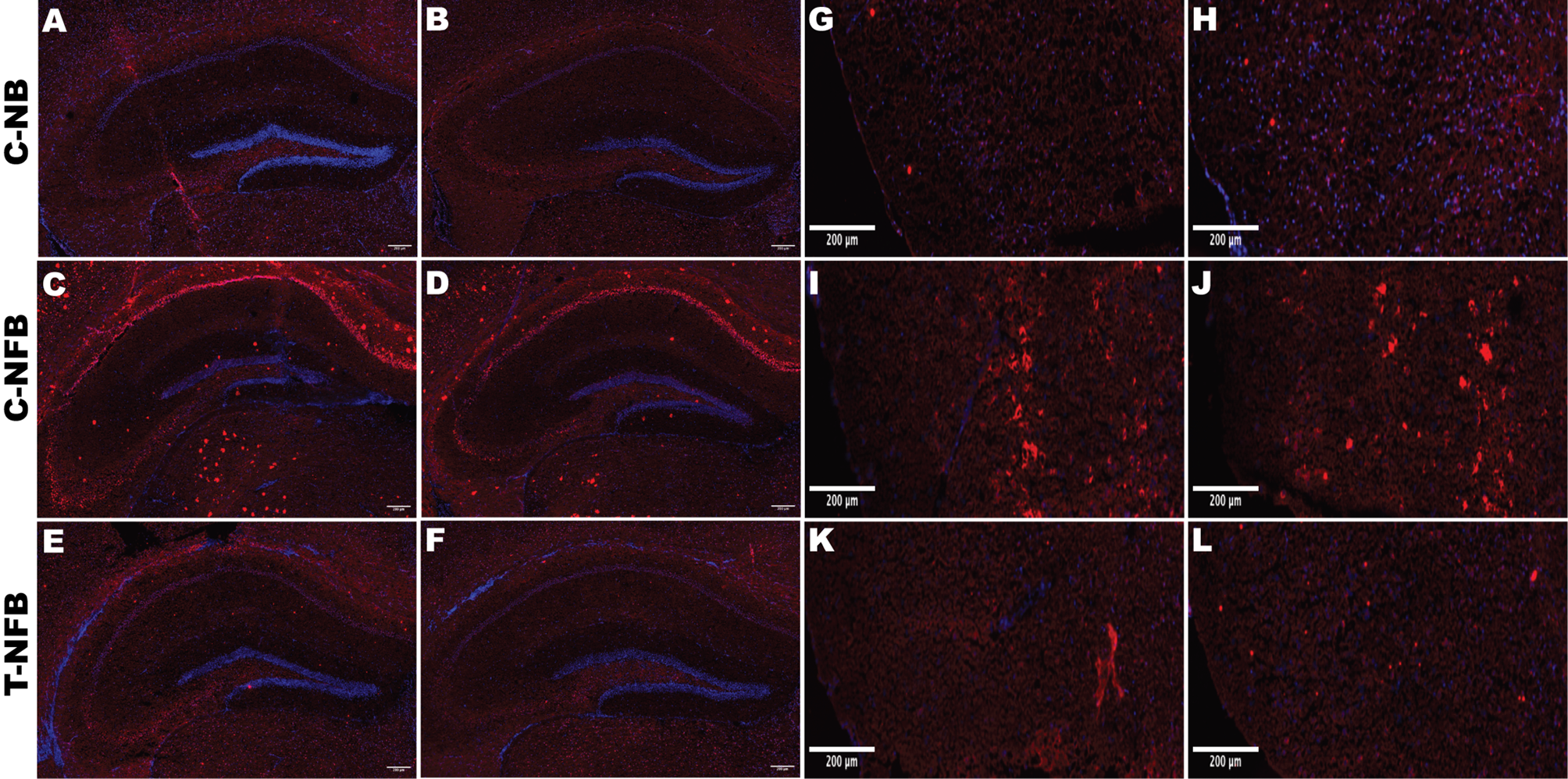

Supplement: Supplementary file 11 [file Image_7.tif]
