# Supplementary material for: Neurogenesis drives hippocampal formation-wide spatial transcription alterations in health and Alzheimer's disease
Source: Front Dement. 2025 Apr 16;4:1546433. doi: 10.3389/frdem.2025.1546433 (PMC12041076; doi:10.3389/frdem.2025.1546433)
Supplement: Supplementary file 12 [file Data_Sheet_1.pdf]

Supplemental Information: "Neurogenesis drives hippocampal formation-wide alterations in health and Alzheimer's disease"

Zachery D. Morrissey[1,2,3], Pavan Kumar[3], Trongha X. Phan[3], Mark Maienschein-Cline[4], Alex Leow[2,5], and Orly Lazarov[3,*]

[1]Graduate Program in Neuroscience, University of Illinois Chicago
[2]Dept. of Psychiatry, University of Illinois Chicago
[3]Dept. of Anatomy & Cell Biology, University of Illinois Chicago
[4]Research Informatics Core, University of Illinois Chicago
[5]Dept. of Biomedical Engineering, University of Illinois Chicago
[*]olazarov@uic.edu

Supplemental Methods

Immunohistochemistry of App

Brain sections from the same animals were used for (10 µm thick fresh frozen) immunofluorescent staining. Sections were first thawed at room temperature for 15 min, and a hydrophobic barrier was created around the section to avoid a spill of incubated solution. Sections were washed with TBST to remove the cryo-mount and treated with freshly prepared 4% cold PFA solution for 5 min, followed by three TBS wash 5 min each. Sections were blocked with five percent normal donkey serum for an hour, followed by primary antibody (1:500; Anti Amyloid precursor protein, A8717-Sigma; RRID: AB_2769499) incubation for three days at 40 °C. Sections were washed with TBST three times, 5 min each. Donkey anti Rabbit secondary antibody was used with 1:500 dilution (Cat- 711-165-152, Jackson laboratories, RRID: AB_2307443). Sections were incubated for 3 h at room temperature with secondary antibody, followed by three wash cycles, 5 min each, with TBS. Finally, sections were treated with DAPI (1:10000) for 5 min, washed with TBST, and mounted with mounting media.

Supplemental Figure Legends

Fig. S1 Statistics for spatial transcriptomics data. (a) Representative scatter plot of cells in the CA1, CA2, CA3, dentate gyrus (DG) and entorhinal cortex (EC). Scale bar indicates 1 mm. (b) Number of cells per mm^2 in each region. Points represent individual mice. Bars represent mean. Error bars represent standard error of the mean (SEM). Blue: C–NB; pink: T–NB; orange: C–NBF; green: T–NBF. (c) Area of DG for each hemisphere. Bars represent mean. Error bars represent SEM. (d) Descriptive statistics for each gene. Left columns indicate the number of spots for each gene. Numbers inside bar represent raw counts. Bars represent log-transformed counts. Right columns represent the relative count of transcripts by group (C–NB, T–NB, C–NBF, T–NBF). Blue: C–NB; pink: T–NB; orange: C–NBF; green: T–NBF. Vertical dashed lines represent even proportions between groups

Fig. S2 UMAP and anatomical plot of binned count data. (a) UMAP embedding of binned count data where color indicates group (left) and individual subject (right). (b) Anatomical scatter plot of each bin point for each subject. Each point represents a $50\,\mu m^2$ patch of tissue. (d–h) Top ten most highly expressed genes for each region. (i) UMAP scatter plot of union top ten most highly expressed genes. Color indicates normalized log1p expression.

Fig. S3. Gene expression profile by region and cell type. (a) Heatmap of (row) z-score relative expression of each gene by region. (b) Heatmap of (row) z-score percent expression of each gene in each cell type. Grayscale values represent average percent expression of each gene across all cell types.

Fig. S4. Neuron differential gene expression. Additional volcano plots of differentially expressed genes for all neurons (a), and excitatory neurons (b) for C–NB/C–NBF and T–NBF/C–NBF comparisons. Differentially expressed genes calculated using Fisher's exact test.

Fig. S5. Glia differential gene expression. Additional volcano plots of differential gene expression for microglia (a), astrocytes (b), and oligodendrocytes (c) for C–NB/C–NBF and T–NBF/C–NBF comparisons. Differentially expressed genes calculated using Fisher's exact test.

Fig. S6. T–NB/C–NB differential gene expression by region and cell type. Volcano plots of differential gene expression in T–NB/C–NB for astrocytes, excitatory neurons, inhibitory neurons, microglia, and oligodendrocytes for each region. Differentially expressed genes calculated using Fisher's exact test.

Fig. S7. Immunohistochemistry of App. Confocal images of brain sections of C–NB (A, B, G, H), C–NBF (C, D, I, J) and T–NBF (E, F, K, L) mice were immunostained with antibodies raised against APP. Images of the hippocampus (A–F) and the lateral entorhinal cortex (G–L). Sections were counterstained with DAPI.

Table S1. Cell counts for each subject, region, and cell type.

Table S2. Cell density statistics for each cell type. For each cell type, a one-way ANOVA was performed to test for differences in the number of cells per mm^2 across groups.

Table S3. Linear regression statistics for C–NB/C–NBF versus T–NBF/C–NBF $\log_2(FC)$ values for each gene.

Table S4. Fisher's exact test of inhibitory neuron proportion for each region and pairwise comparison.
